# Experimentally Assessing the Electronic Structure and Spin-State Energetics in MnFe Dimers Using 1s3p Resonant Inelastic X-ray Scattering

**DOI:** 10.1021/acs.inorgchem.4c01538

**Published:** 2024-09-16

**Authors:** Rebeca G. Castillo, Benjamin E. Van Kuiken, Thomas Weyhermüller, Serena DeBeer

**Affiliations:** † Max Planck Institute for Chemical Energy Conversion, Stiftstrasse 34, Mülheim an der Ruhr D-45470, Germany; ‡ Laboratory of Ultrafast Spectroscopy (LSU) and Lausanne Centre for Ultrafast Science, École Polytechnique Fédérale de Lausanne (EPFL), Lausanne CH-1015, Switzerland; § European XFEL, Holzkoppel 4, Schenefeld D-22869, Germany

## Abstract

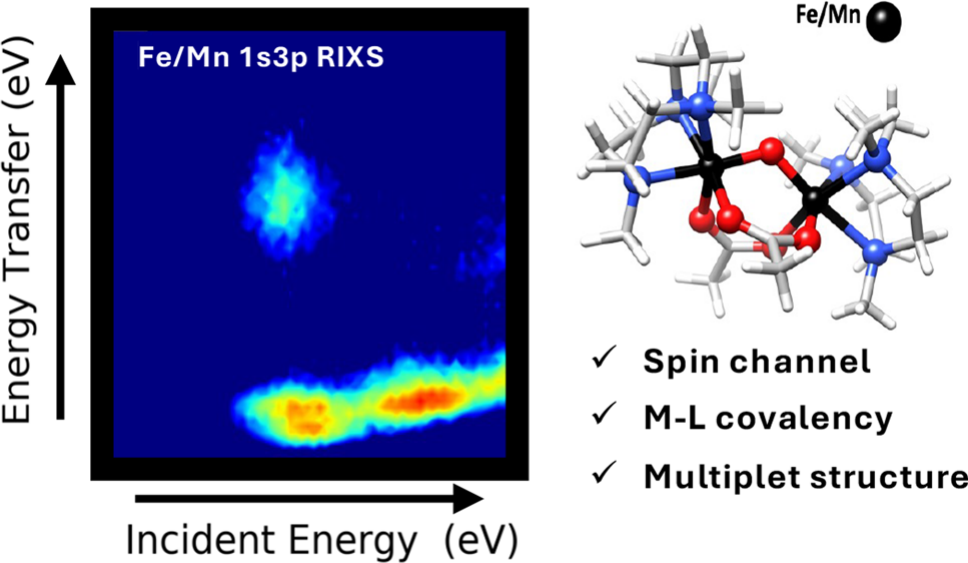

The synergistic interaction between Mn and Fe centers is investigated via a comprehensive analysis of full 1s3p resonant inelastic X-ray scattering (RIXS) planes at both the Fe and Mn K-edges in a series of homo- and heterometallic molecular systems. Deconvolution of the experimental two-dimensional 1s3p RIXS maps provides insights into the modulation of metal–ligand covalency and variations in the metal multiplet structure induced by subtle electronic structural differences imposed by the presence of the second metal. These modulations in the electronic structure are key toward understanding the reactivity of biological systems with active sites that require heterometallic centers, including MnFe purple acid phosphatases and MnFe ribonucleotide reductases. Herein, we demonstrate the capabilities of 1s3p RIXS to provide information on the excited state energetics in both element- and spin-selective fashion. The contributing excited states are identified and isolated by their multiplicity and π- and σ-contributions, building a conceptual bridge between the electronic structures of metal centers and their reactivity. The ability of the presented 1s3p RIXS methodology to address fundamental questions in transition metal catalysis reactivity is highlighted.

## Abstract 2

In both biological and chemical catalysis, the synergistic interaction between two metals is often important for optimizing activity. This work investigates this phenomenon in Mn and Fe homo- and heterometallic molecular systems using 1s3p resonant inelastic X-ray scattering (RIXS). We provide a detailed description of the chemical information contained in the 1s3p RIXS plane, and it is shown to be a spin-selective probe of the low-lying excited state energetics.

## Introduction

Iron and manganese play key roles in reactivity in both biological and chemical catalysis.^1−10^ In nature, Fe-cofactors are well-known for performing oxygen reduction and transport,^11−17^ while the Mn-cofactor in the oxygen-evolving complex of photosystem II enables O_2_ production.^18−21^ While in the aforementioned cases, Fe and Mn play very specific roles, in other cases, biology utilizes Mn and Fe to fulfill common roles. This is the case for both superoxide dismutases^22^ and homoprotocatechuate 2,3-dioxygenases,^23^ where the latter shows essentially identical reactivity with either Mn or Fe at the active site.

In other cases, it is clear that biology optimizes the active sites for a very specific metal composition.^24−26^ For instance, FeMn purple acid phosphatases and FeMn ribonucleotide reductases (RNRs) have been identified in which optimal enzymatic activity is only achieved when the Fe to Mn ratio is 1:1.^27^ Given the similar coordination environments surrounding the Mn and Fe binding sites, the observed selectivity in these enzymes is remarkable and whether the metal binding is driven by thermodynamic or kinetic control remains an open question.^24,27,28^ Regardless, nature’s choice of two different metals at the active site suggests some added synergy as a consequence of the resultant electronic structure that is not found in the analogous FeFe or MnMn structures.

We note that there are numerous other examples of enzymes with heterometallic cofactors, including NiFe hydrogenases,^29^ Cu/Zn superoxide disumatases,^30^ the Cu/Fe site of cytochrome *c* oxidase,^31^ and the complex FeMco factors of nitrogenase (where M = V or Mo).^32,33^ Furthermore, there are countless examples in heterogeneous catalysis, where the addition of a second metal greatly enhances the catalytic activity. These include the addition of iron and cobalt to Ni-based water oxidation^34^ and dry methane reforming catalysts, respectively, to name only a few examples.^35,36^ Taken together, these examples strongly suggest that there is a need to deepen our understanding of the synergistic interaction of metals in heterometallic active sites and how they differ from those in homometallic active sites.

It is clear that in this vein, element selective X-ray spectroscopic approaches have played an important role, allowing each metal in a complex catalytic active site to be addressed separately.^37−42^ For MnFe RNR specifically, two color X-ray emission spectroscopic (XES) measurements have been utilized to evaluate the changes that occur at both Mn and Fe as a function of oxidation state.^43^ Unfortunately, the relatively modest resolution (due to the 1s core hole lifetime broadening), together with the potentially canceling effects of covalency and metal d-count, greatly limits the information content of these spectra.^44−46^ Similarly, conventional K-edge X-ray absorption spectra (XAS) also suffer from large core hole lifetime broadenings, which again limit the spectral resolution and, ultimately, the information content.^47−49^ Importantly, neither K-edge XAS nor XES alone allows one to readily assess the energetics of the low-lying excited state multiplets, which are key to reactivity.

The resonant inelastic X-ray scattering (RIXS) (also known as resonant XES or RXES) approach utilized herein is a photon-in/photon-out experiment where the XES spectrum is monitored for a particular resonant excitation of a core electron.^47,50,51^ When this process involves 1s (K-edge) absorption followed by a 3p to 1s emission process, it is known as 1s3p RIXS.

**Figure 1:**
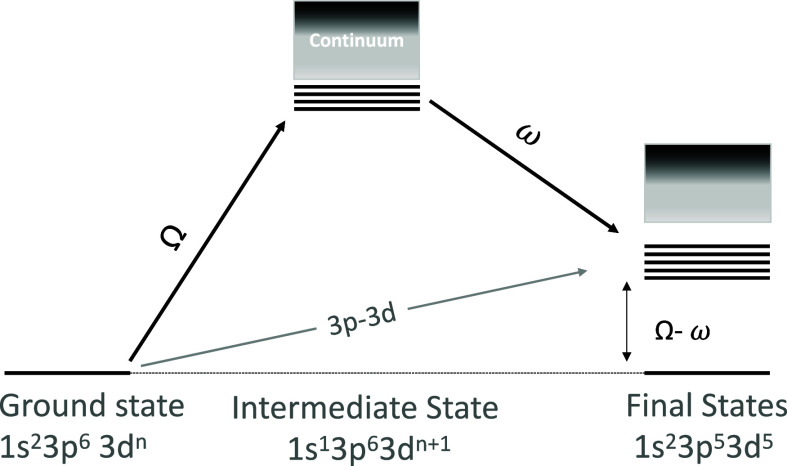
Schematic of the 1s3p RIXS process, including the electronic configurations for the ground state, intermediate states upon excitation into the 3d manifold, and the corresponding final states.

A simple scheme of the 1s3p RIXS process is shown in Figure 1 for the case when the excitation energy is tuned to the pre-edge (1s → 3d) region of a 3d transition metal K-edge XAS spectrum. A 1s^2^3p^6^3d^*n*^ ground state is excited to a 1s^1^3p^6^3d^*n*+1^ intermediate state configuration upon the absorption of an X-ray photon. In the subsequent X-ray emission step, a 3p → 1s transition yields the 1s^2^3p^5^3d^*n*+1^ final state. The difference between the initial and final state energies is referred to as the energy transfer, and in the case of 1s3p RIXS, one arrives at an M-edge like (3p→ 3d) final state.

The 1s3p RIXS process is theoretically described by the Kramers–Heisenberg (KH) equation, as shown below (eq 1)^52^
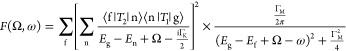
1with ground (g), intermediate (n), and final (f) state energies *E*_g_, *E*_n_, and *E*_f_, and transition operators *T*_1_ and *T*_2_ for absorption and emission processes, respectively. Intermediate and final state lifetime broadenings for the 1s3p RIXS process are given by Γ_K_ and Γ_M_, respectively. The incident and emitted (ω) X-ray energies are given, respectively, by Ω and ω.

Perhaps the most frequent use of RIXS in the hard X-ray regime is the application of 1s2p RIXS to obtain so-called high-energy resolution fluorescence detected (HERFD) XAS spectra.^53−58^ This is most typically performed by taking a cut of a 1s2p RIXS plane at a constant emission energy. This approach has been particularly effective for obtaining high-resolution pre-edge data for further quantitative correlation with theory^55,59−61^ and also for the elimination of metallic background scattering signals.^62^ In addition, 1s2p RIXS has been utilized to obtain L-edge-like information utilizing a hard X-ray probe.^50,63−67^ In contrast, 1s3p RIXS has been relatively far less explored, though notably it has been utilized to obtain spin and/or site selective XAS,^68−73^ and most recently, we have demonstrated its utility as a means to recover oxidation state information that may be lost in conventional X-ray spectroscopy.^74^ However, the full information content of the entire 1s3p RIXS planes is still being developed,^75−77^ and its use to assign the identity of low lying excited state multiplets is thus far relatively unexplored.^78^ Moreover, to our knowledge, the detailed electronic structure content of 1s3p RIXS planes has not been previously applied to understand heterometallic systems.

**Figure 2:**
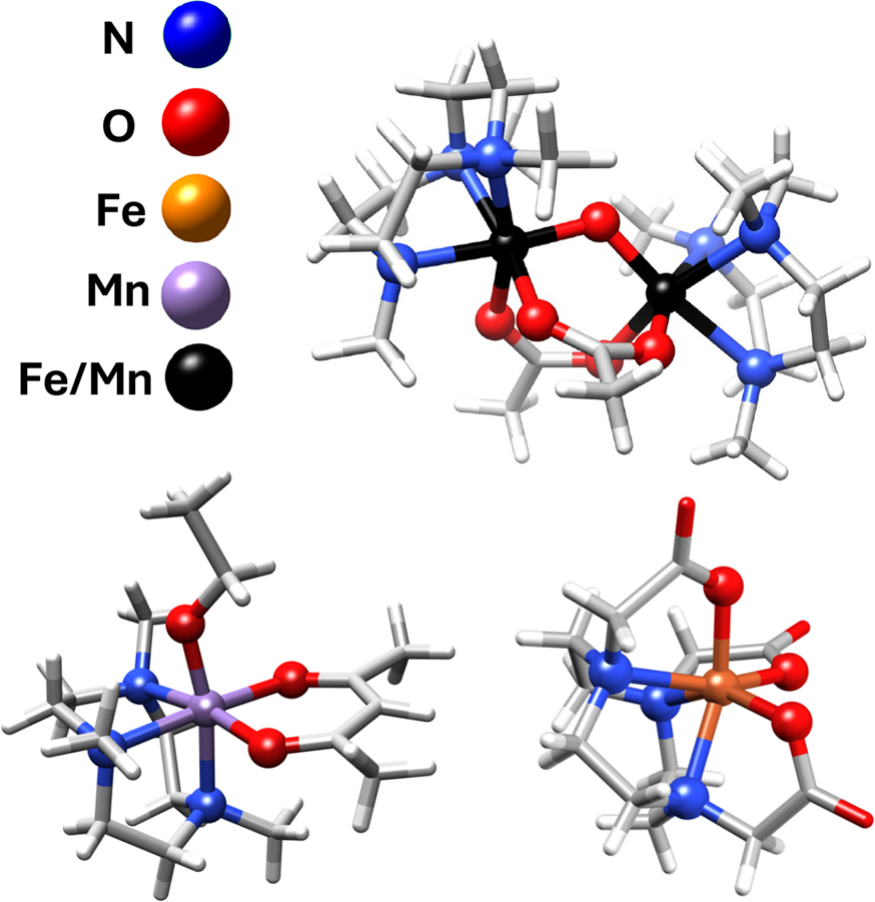
Metal geometry for all monomeric and dimeric model complexes including all Fe–N/O and Mn–N/O bonds.

To this end, we have been interested in fully developing an interpretive framework for 1s3p RIXS to probe heterometallic active sites.^79^ Herein, we specifically focus on a series of FeFe, MnMn, and MnFe dimers, together with related monomers, in order to systematically evaluate the subtle changes in the electronic structure of homometallic versus heterometallic complexes that may be hidden, or difficult to assess, in standard XAS and XES measurements. The series of complexes that we have selected share similar geometries and ligand environments. The metal dimers have one oxo and two acetate bridges (see Figure 2) and span a combination of metals and oxidation states including Fe(III)Fe(III), Mn(III)Mn(III), Mn(III)Fe(III), and Mn(IV)Fe(III).^80−84^ All dimers are antiferromagnetic coupled with the exception of the Mn(III)Mn(III). Additionally, Fe(III) and Mn(III) high-spin monomers were included to assess the effect of the second metal atom. The key bond distances, as well as total (*S*_t_) and local (*S*_loc_) spins, for all models are provided in Table 1. By selectively monitoring the electronic structure of both metal sites in heterometallic systems, a more detailed description of the subtle electronic structure modulations induced by a second metal can be obtained. Hence, the present studies establish a route toward understanding the synergistic interaction between Mn and Fe in biological systems and also provide tools that should be generally transferable to a wide range of homo- and heterometallic catalysts.

**Table 1:** M–M and M–O Bond Lengths and Total and Local Spin States for the Studied Model Complexes

complex	M–M (Å)	Fe–O (Å)	Mn–O (Å)	*S*_t_ (*S*_loc_)
FeIIIFeIII	3.15	1.8		0 (5/2)
MnIIIMnIII	3.12		1.81	4 (4/2)
MnIIIFeIII	3.12	1.82	1.78	1/2 (4/2,5/2)
MnIVFeIII	3.20	1.80	1.81	1 (3/2,5/2)
FeIII		1.98/2.18		5/2
MnIII			1.79/2.02	4/2

## Methods

### Sample Preparation

All model complexes used in this work were synthesized following previously reported procedures.^80−82,84^ Each model complex is labeled by its metal oxidation state as follows: [FeIII(TCTA)] as **FeIII**, [ttacnMn(III)(acac)(OC_2_H_5_)]^1+^ as **MnIII**, [(ttacn)_2_Mn(III)_2_(μ-O)(mu-OAc)_2_]^2+^ as **MnIIIMnIII**, [(ttacn)_2_Fe(III)_2_(μ-O)(μ-OAc)_2_]^2+^ as **FeIIIFeIII**, [(ttacn)_2_Fe(III)(μ-O)(μ-OAc)_2_Mn(III)]^2+^ as **MnIIIFeIII**, [(ttacn)_2_Fe(III)(μ-O)(μ-OAc)_2_Mn(IV)]^3+^ as **MnIVFeIII**—with TCTA being 1,4,7-triazacyclononane-*N*,*N*′,*N*″-triacetate and ttacn being *N*,*N*′,*N*″-trimethyl-1,4,7-triazacyclononane. All samples were diluted with boron nitride and packed into aluminum spacers sealed with 38 μm Kapton tape windows.

### Data Collection

All experimental data were measured at beamline ID26 at the European Synchrotron Radiation Facility (ESRF). The ESRF storage ring was operating at 6 GeV, and the experiment was performed during a 7/8 + 1 filling mode with 200 mA current. A Si(311) double crystal monochromator was used for the selection of the incident X-ray energy instead of Si(111) due to its larger resolving power (*E*/Δ*E* > 35,000). The incident flux was >10^13^ photons/s, with beam size at the sample of 0.1 mm (v) × 1 mm (h). Calibration of the incident energy for Fe K-edge experiments was performed by setting the first inflection point of an Fe foil to 7111.2 eV.^85,86^ Calibration of the incident energy for Mn K-edge experiments was carried out by using KMnO_4_ and setting the pre-edge maxima at 6543.3 eV, as performed in previous Mn studies.^87,88^ X-ray emission was measured by utilizing a Johann-type spectrometer consisting of a set of five spherically bent analyzer crystals aligned in a Rowland geometry and a silicon drift detector. Analyzer crystals selected for Fe and Mn Kβ XES detection were Ge(620) and Si(440), respectively. The energy dispersion was calibrated at the beamline to the scatter peaks, and the absolute energy calibration was determined by shifting the spectra to align with the Fe Kβ emission features of Fe_2_O_3_ (Kβ_1,3_ = 7060.6 eV and Kβ′ = 7045.2 eV) and the Mn Kβ emission features of Mn_2_O_3_ (Kβ_1,3_ = 6490.4 eV). The overall experimental resolution for resonant X-ray emission was 1.1–1.2 eV, estimated from the full width at half-maximum of the elastic peak measured at the Fe and Mn Kβ XES mainline. These values are the convolution of the spectrometer (1.08 eV for Fe and 1.18 eV for Mn) and monochromator (0.2 eV) bandwidths. Samples were maintained at temperatures of 20 K, using a liquid helium flow cryostat. Detailed damage studies were performed on each sample for both Fe and Mn experiments by collecting short XAS spectra at different dwell times. Attenuation of the incident beam was necessary for all of the Mn compounds. This was achieved by inserting aluminum foil into the beam path to attenuate 60–70% of the incoming beam. Although no attenuation of the flux appeared to be necessary for the Fe measurements, in the heterometallic complexes, the same attenuation selected for the Mn site was used.

For all complexes, nonresonant Kβ mainlines were first measured, followed by Kβ_1,3_ HERFD-XAS as a first check for oxidation states and to evaluate if any differences between models with the same metal oxidation state were observed. These measurements also served as a reference to evaluate a sensible range for measurement of 1s3p RIXS planes. Nonresonant Kβ mainlines were measured over a range of 6460–6506 and 7025–7075 eV for Mn and Fe, respectively, with a 0.15 eV energy step, and data were normalized to the maximum of the emission intensity. Kβ_1,3_ HERFD-XAS spectra were measured over a range of 6535–6595 and 7106–7175 eV for Mn and Fe, respectively, using an energy step size of 0.1 eV in both cases. Longer HERFD-XAS scans (6496–6840 and 7000–7920 eV for Mn and Fe, respectively) were measured to facilitate data pre- and post-edge background subtraction. All 1s3p RIXS planes were obtained by measuring HERFD-XAS spectra at different emission energies and using an energy step size of 0.15 eV for the detected emission energy.

### Computational Details

Multiplet simulations were performed using the EDRIXS set of python modules extended locally with tools for computing XES spectra.^89^ Both nonresonant Kβ XES and 1s3p RIXS spectra were calculated in the framework of atomic multiplet theory, as recently reported.^74^ Calculations included 1s, 3p, and 3d atomic shells. The two-electron integrals were parametrized by the commonly used Slater–Condon factors, and the ligand field is considered in *O*_*h*_ symmetry. A 10Dq value of 2 eV was chosen based on the energy separation of the pre-edge features and the comparisons of experimental and simulated data for nonresonant XES. The Slater–Condon parameters were scaled to account for the differences in covalency of the studied systems, using 60–65% scaling. For both Mn and Fe RIXS simulations, the configurations for the initial, intermediate, and final states were chosen as 1s^2^3p^6^3d^*n*^, 1s^1^3p^6^3d^*n*+1^, and 1s^2^3p^5^3d^*n*+1^, respectively.

Term assignments were made by analyzing the states involved in the RIXS process. The simulations yield a complete set of state energies and wave functions for the initial, intermediate, and final states. These are utilized to compute the expectation values of the angular momentum operators L_*i*_, S_*i*_, and J_*i*_ (*i* = *x*, *y*, *z*) and their squares S^2^, L^2^, and J^2^. By analyzing the degeneracy, angular momentum, and spin properties of the states, term symbols are ascribed. Furthermore, we work in the limit where no SOC is included, which greatly clarifies the state identity.

## Results and Analysis

### Metal K-Edge XAS and Nonresonant XES

**Figure 3:**
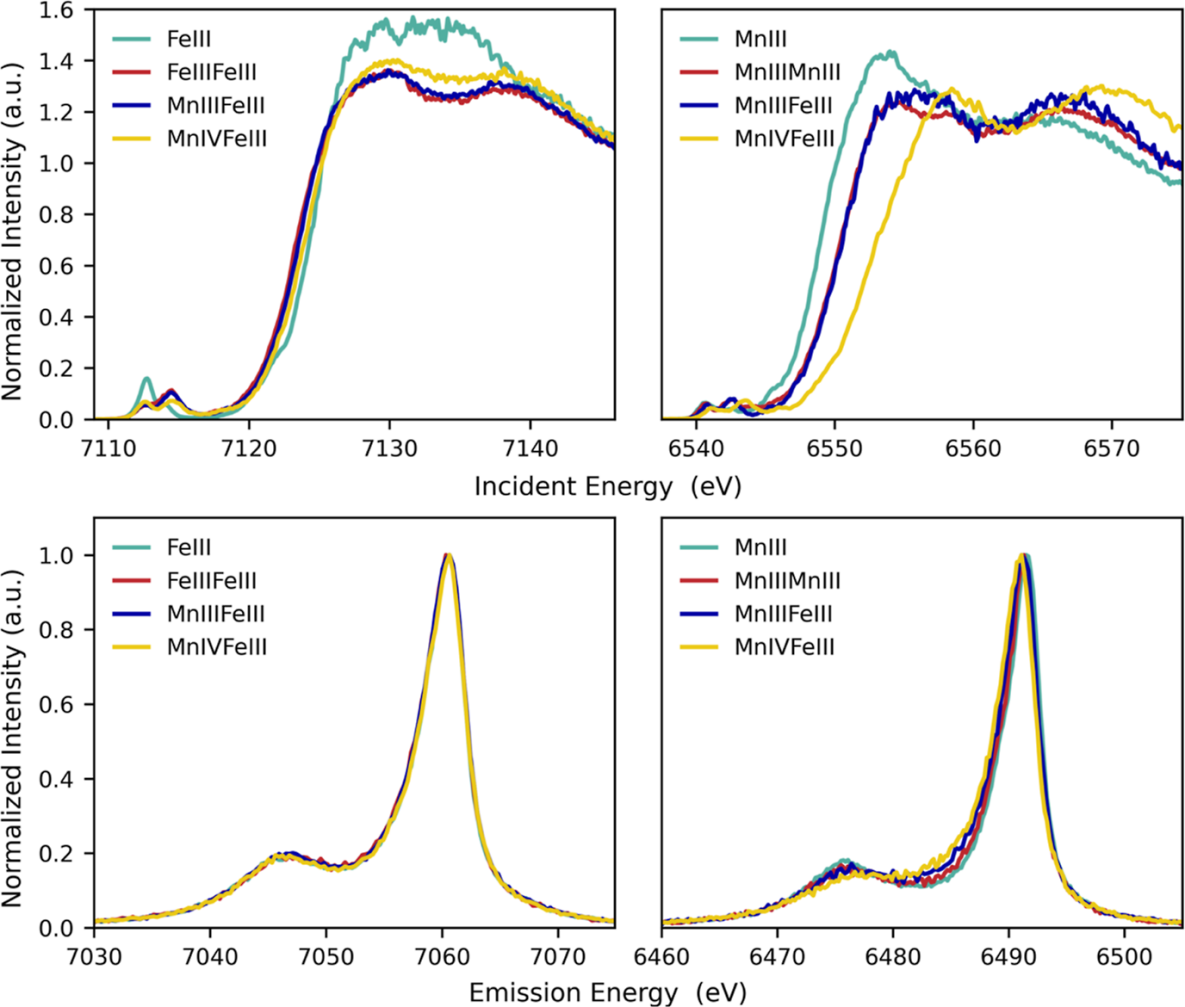
Fe (left) and Mn (right) Kβ_1,3_-HERFD XAS (top) and nonresonant Kβ XES (bottom) for all model complexes.

Before the more complex 1s3p RIXS spectra were evaluated, the Fe and Mn Kβ_1,3_ HERFD-XAS and Kβ mainline XES for all model complexes are briefly presented. Figure 3 (top) shows the Fe (left) and Mn (right) Kβ_1,3_ HERFD XAS of all model complexes. In all cases, the constant emission energy chosen for the Kβ_1,3_ HERFD XAS corresponds to the maxima of the Kβ_1,3_ nonresonant XES. The Fe spectra show small differences for all models relative to the Mn spectra, where variations in the edge position are more significant. This observation for the Fe K-edge is expected since all iron-containing models have a high-spin ferric ion. On the other hand, the Mn Kβ_1,3_ HERFD-XAS shows that the rising edge of the MnIII monomer appears ∼1.5 eV below that of the MnIIIMnIII and MnIIIFeIII dimers despite possessing identical local oxidation and spin states. This is a clear example of how difficult it can be to extract physical oxidation state information when covalency increases.^45,90^ In a study of molecular iron complexes, we have previously shown how resonant emission measurements can overcome such ambiguities in oxidation state assignments. This is partially because in resonant XES, one obtains higher resolution than in the nonresonant limit. Additionally, by resonantly exciting at the pre-edge, the 3p-to-1s XES decay process will result in a different final state multiplet compared to nonresonant XES. Taken together, these factors make resonant XES more sensitive to changes in the multiplet structure of different oxidation states.^74^ Finally, the MnIVFeIII appears at 1.9 eV higher in energy than Mn(III)-containing dimers, as expected due to the increase of the Mn oxidation state.

Figure 3 shows the Fe (left) and Mn (right) Kβ XES mainlines of all models. Both the Fe and Mn spectra are comprised of two main features known as the Kβ′ at the low-energy side of the spectra and the Kβ_1,3_ at the high-energy side. These features are split by ∼14.1–14.5 eV for Fe Kβ XES and 13.9–15.7 eV for Mn Kβ XES (see Table S2 for tabulated values). As in the HERFD-XAS spectra, the Kβ XES mainlines show greater similarity in the Fe measurements than in the Mn measurements. Kβ XES mainlines capture the nonresonant emission process occurring when the metal 1s core hole is filled by an electron from the metal 3p manifold. These dipole-allowed transitions in this case (Δ*L* = +1) result in a 1s^2^3p^5^3d^*n*^ final electronic configuration through the 3p → 1s decay.

The Kβ_1,3_ and Kβ′ features split as a consequence of the 3p-3d exchange contribution to the 1s^2^3p^5^3d^*n*^ final state energy,^91−94^ which differs depending on whether an alpha (α) or beta (β) core hole is created during the photoionization. The two possibilities of the *m*_s_ value of the photoionized electron yield total multiplicities for the final states [2(*S*_(3d-shell)_ ± 1/2_(3p-shell)_) + 1]. Most of the final states associated with the Kβ_1,3_ feature have the higher multiplicity (2(*S*_(3d-local)_ + 1/2_(3p-local)_) + 1), while the Kβ′ feature is dominated by the lower total multiplicity [2(*S*_(3d-local)_ – 1/2_(3p-local)_) + 1]. This is referred to as 3p-3d exchange splitting, and it is the main reason why this technique is sensitive to the metal oxidation state and spin state. Thus, the constant +3 oxidation state, similar covalency, and 5/2 spin state on all the Fe sites result in the nearly superimposable Fe Kβ XES mainlines for all models, in which the Kβ_1,3_ and Kβ′ features split by ∼3 eV per unpaired electron. The final states of Kβ XES mainline spectra can be analyzed by atomic Russell–Saunders terms (Table S1)^95^ because inclusion of ligand field does not greatly affect these spectra in the high-spin limit.^44,94^ For Fe(III) HS, the dipole-allowed 3p to 1s emission process gives rise to Kβ_1,3_ and Kβ′ features, which correspond to ^7^P and ^5^P terms, respectively.^44,93,96^

The energy splitting of the Mn Kβ mainlines for Mn(III)-containing models is ∼15.3–15.7 eV, while the splitting for MnIVFeIII decreases to 13.9 eV, and the intensity of the Kβ′ feature also decreases. This is expected as all Mn(III)-containing models have the same oxidation state and spin state (*S* = 2), while in MnIVFeIII, the Mn d^*n*^-count and 3d local spin state have decreased by one unit (*S* = 3/2). Here, the Mn(III) reaches ^6^D and ^4^D states upon ionization of a β or α 1s electron, respectively, and thus the final states after the 3p electron refills the 1s core hole are also divided into sextets and quartets, which define the Kβ_1,3_ and Kβ′ features, respectively, as summarized in Table S2. The Mn(IV) has a spin of 3/2 and reaches ^5^F and ^3^F states upon 1s ionization, which, followed by 3p to 1s decay, results in a set of final states that split dominantly into quintets (Kβ_1,3_) and triplets (Kβ′) (Table S2). Overall, the nonresonant Kβ XES data presented in Figure 3 serve as reasonable fingerprints for oxidation state and spin states. In the case of the models that share the same oxidation state and have similar total covalency, the nonresonant Kβ XES contains no additional information, thus limiting its ability to probe more subtle electronic structural changes.

### XAS Pre-Edges

**Figure 4:**
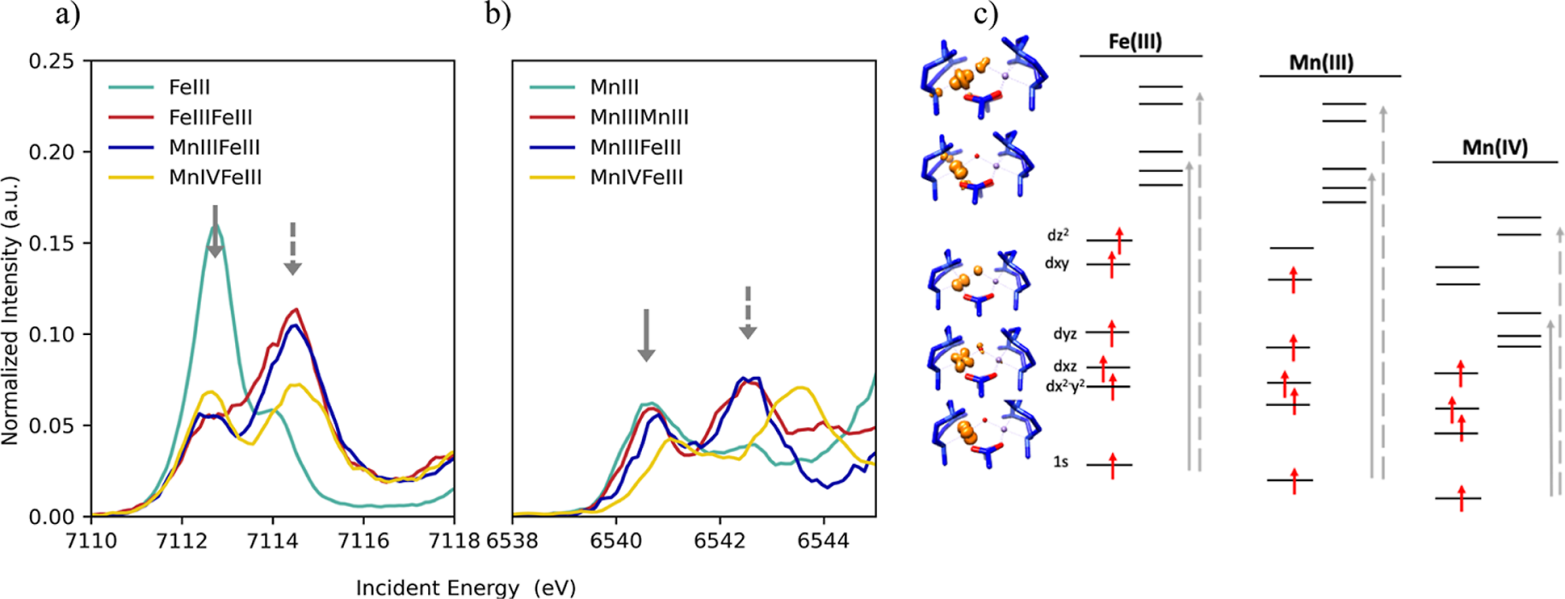
Fe (a) and Mn (b) pre-edge regions of Kβ_1,3_-HERFD XAS spectra (taken by selecting the maximum of the nonresonant XES spectra for each model at fixed emission energy) and (c) diagram of 1s → 3d transitions in pseudo *O*_*h*_ environment for Fe(III), Mn(III), and Mn(IV) via β spin channel marked by gray arrows (solid for transitions into t_2g_-like orbitals and dashed for transitions into e_g_-like orbitals).

In this section, the information contained in the XAS spectra is further investigated with a detailed analysis of the pre-edge region of the spectra. Figure 4a,b shows an expansion of the pre-edge region for the Fe and Mn XAS spectrum, respectively, which is formally comprised of quadrupole transitions (Δ*L* = ±2) from the metal 1s to unoccupied 3d orbitals. The pre-edge intensity can be significantly enhanced by dipole contributions, which result from 4p mixing into the 3d orbitals when the symmetry at the metal site decreases.^86^ While Kβ_1,3_-HERFD XAS has a sharper pre-edge than conventional XAS, not all 1s → 3d transitions are captured. This is due to the spin selectivity of Kβ_1,3_-HERFD XAS. Conventional XAS pre-edges (often collected in transmission or total fluorescence yield mode) contain both alpha (α) and beta (β) 1s-3d transitions. As explained in the analysis of the Kβ XES results above, the Kβ_1,3_ feature is comprised of the final states resulting from β transitions. Thus, the Kβ_1,3_ HERFD-XAS pre-edge region gives preference to the β-channel, and therefore, the α transitions are not captured. This is shown in Figure 4c, where only the β-channel transitions are displayed.

Both Fe and Mn exhibit two features in the pre-edge region split by ∼1.8–2.5 eV for the dimers and ∼1.4 and ∼1.7 eV for Fe and Mn monomers, respectively (see Table S3 for tabulated splitting values and IWAEs). The absolute energy positions of the spectra are observed to be a function of the metal oxidation state, which is nearly constant for the Fe dimers, but for the Mn models, the spectrum for the MnIVFeIII dimer is found to be shifted by ∼1.5 eV higher energy relative to all MnIII spectra, consistent with the increase in Mn oxidation state. There are also notable changes in the intensity ratios of the two pre-edge features for different compounds.

In the case of the Fe pre-edges, the intensity ratio is significantly different between the monomer and dimers with the monomer being dominated by a more intense low-energy feature and the Fe(III)- and Mn(III)-containing dimers having a more intense high-energy peak. In contrast, the MnIVFeIII dimer shows two pre-edge features of roughly equal intensity.

In the case of the Mn pre-edges, the monomer exhibits a similar intensity low-energy feature in comparison to the dimers. As for the Fe pre-edges, the homometallic (MnIIIMnIII) and heterometallic (MnIIIFeIII) dimers have a similar Mn pre-edge, although the intensity of the low-energy feature is somewhat decreased for the heterometallic complex. Finally, the energy separation of the MnIVFeIII pre-edge peaks is the largest (∼2.5 eV), indicating that the local ligand field splitting at the Mn has increased relative to the Mn(III)-containing models (with pre-edge splitting of ∼1.7–1.8 eV), and the intensity of the low-energy feature has decreased in comparison with both MnIIIMnIII and MnIIIFeIII.

The origin of the two features observed in the pre-edge spectra can be rationalized in a simple 1-electron picture with inclusion of ligand field effects imposed by the pseudo-octahedral environment of all complexes, as shown in Figure 4c. Note these dimers are not rigorously octahedral and none of these molecules have inversion symmetry but they have a *C*_2*v*_ symmetry (with each monomer half having *C*_*s*_ symmetry), their *z* axes are aligned with the M-oxo bridge, and furthermore, the Mn(III) d^4^ imposes a Jahn–Teller distortion.^97^ To simplify the discussion, we will consider a modified *O*_*h*_ case, where 3d orbitals are in energetic order: 3d_*x*_^^2^^_–*y*_^^2^^, 3d_*xz*,_ 3d_*yz*,_ 3d_*xy*,_ 3d_*z*_^^2^^, in order to be consistent with previous literature reports on these complexes.^80,97^ We refer to π-interacting 3d orbitals as the 3d_t_2g__-like set and σ-interacting 3d orbitals as the 3d_e_g__-like set. Following this picture, the first pre-edge peak is due to transitions from the metal 1s to the 3d_t_2g__-like set (Figure 4c, solid gray arrows), and the second peak is due to transitions to the 3d_e_g__-like set (Figure 4c, dashed gray arrows). The energy splitting values of the pre-edge peaks in both cases can be used as an approximate 10Dq value. This energy splitting will be analyzed in more detail in the following sections by the specific states generated in a multiplet framework, including the ligand field splitting.

In both Fe and Mn pre-edges for all dimers, the short distance between the metal site and the mu-oxo bridge (aligned along the *z* axes) induces a higher dipole contribution in 1s → 3d-e_g_ transitions,^98^ and thus an increased intensity of this pre-edge peak is observed relative to the monomers. Upon going from Mn(III) to Mn(IV), there is one additional d-hole, and the metal–ligand bond lengths should decrease. Both of these factors increase the Mn pre-edge intensity. However, the pre-edge intensities do not follow this simple trend because Mn(III) complexes are subject to Jahn–Teller distortions and Mn(IV)-mu oxo bond elongation counteracts the expected increase in bond strength. Furthermore, the presented Kβ_1,3_ HERFD cuts only reflect the β-excitation channels. As both Mn(III) and Mn(IV) have possible 1s to 3d α-excitation channels, a full analysis of all possible excitations requires studying both Kβ_1,3_ and Kβ′ HERFD-XAS pre-edges, which are contained within the 1s3p RIXS plane.

### Fe and Mn 1s3p RIXS Planes

**Figure 5:**
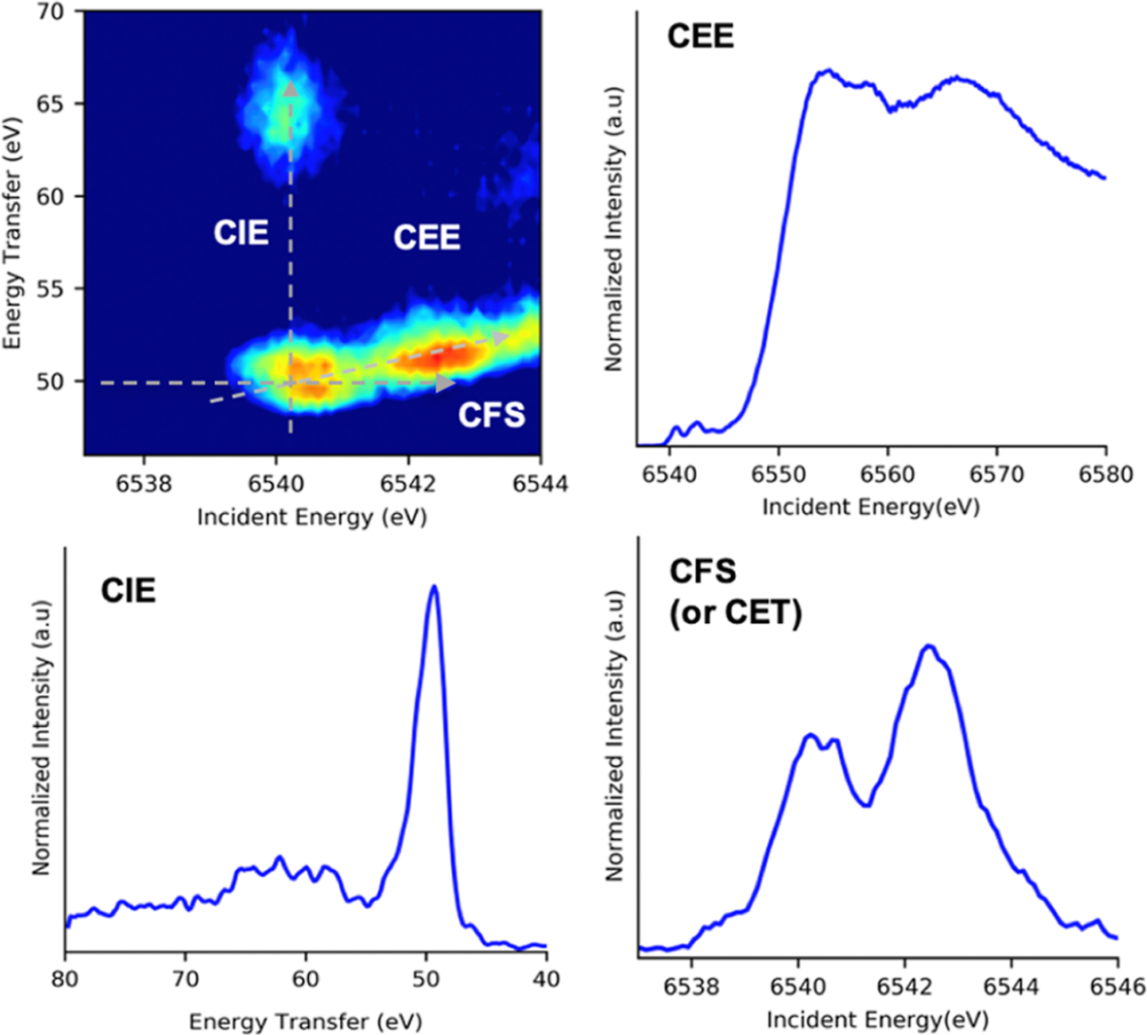
Corresponding CIE, CEE, and CET cuts contained in a 1s3p RIXS plane.

In the following sections, the Fe and Mn 1s3p RIXS planes for all of the models are presented. A description of the 2-dimensional 1s3p RIXS planes and the different cuts that may be taken in order to extract the information encoded in these data is first briefly discussed.^99^Figure 5 presents a representative Mn 1s3p RIXS plane. The spectral content of the RIXS plane can be dissected by generating specific cuts: constant emission energy ω (CEE), constant incident energy Ω (CIE), and constant energy transfer (CET) Ω–ω [also known as constant final state (CFS), ]. Slicing the plane along a CEE corresponds to taking a diagonal of the plane, resulting in a spectrum known as HERFD XAS, as discussed in the previous section. Distinct CEE slices (HERFD XAS spectra) are produced by tuning the emission energy to specific features identified in the nonresonant XES. In the high-spin limit, the CEE slices for transition metal complexes were taken at the maxima of the nonresonant Kβ_1,3_ and Kβ′ emission energies give absorption spectra dominated by β and α transitions, respectively, as explained in the previous section. The second set of slices of the RIXS plane are CIE decompositions, which correspond to the set of final states originating from the isoenergetic intermediate states. When plotting the RIXS plane as incident energy versus energy transfer (Ω–ω) (rather than emission energy), the resulting CIE spectra are the equivalent of M-edge 3p-to-3d excitations. Thus, the M-edge-like final states can be accessed in CIE plots without the need of performing challenging extreme ultraviolet (XUV) experiments in vacuum environments.^100^ Lastly, CET cuts provide a probe of all 1s resonances that decay into a specific final state. It should be noted that different cuts of the RIXS planes will have different intrinsic broadenings. The CET cuts will be dominated by the 1s core hole lifetime broadening Γ_K_, which in the Fe and Mn case corresponds to 1.25 and 1.16 eV, respectively.^101^ On the other hand, the CIE cuts are dominated by the 3p core hole lifetime Γ_M_ broadenings of 1.4 and 1.2 eV for Fe and Mn, respectively.^102^ When the intensity of the slice is integrated over a CET or CIE cut, lifetime broadening arises from the intermediate and final states, previously defined as Γ_K_ and Γ_M_, respectively, in the KH equation. The experimental resolution of the 1s3p RIXS plan is maximized in the CEE slices, taken at 45° from both Γ_K_ and Γ_M_ (Figure 5),^47^ giving a lifetime broadening defined as *f*_v_ by Glatzel and Bergmann^47^ of 0.21 and 0.18 eV for Fe and Mn, respectively. Considering the total experimental broadening, the CEE slides have a total broadening of 1.12 (Fe) and 1.2 eV (Mn). CEE, CIE, and CET broadenings are summarized in the Supporting Information (Table S4).

### Fe 1s3p RIXS

**Figure 6:**
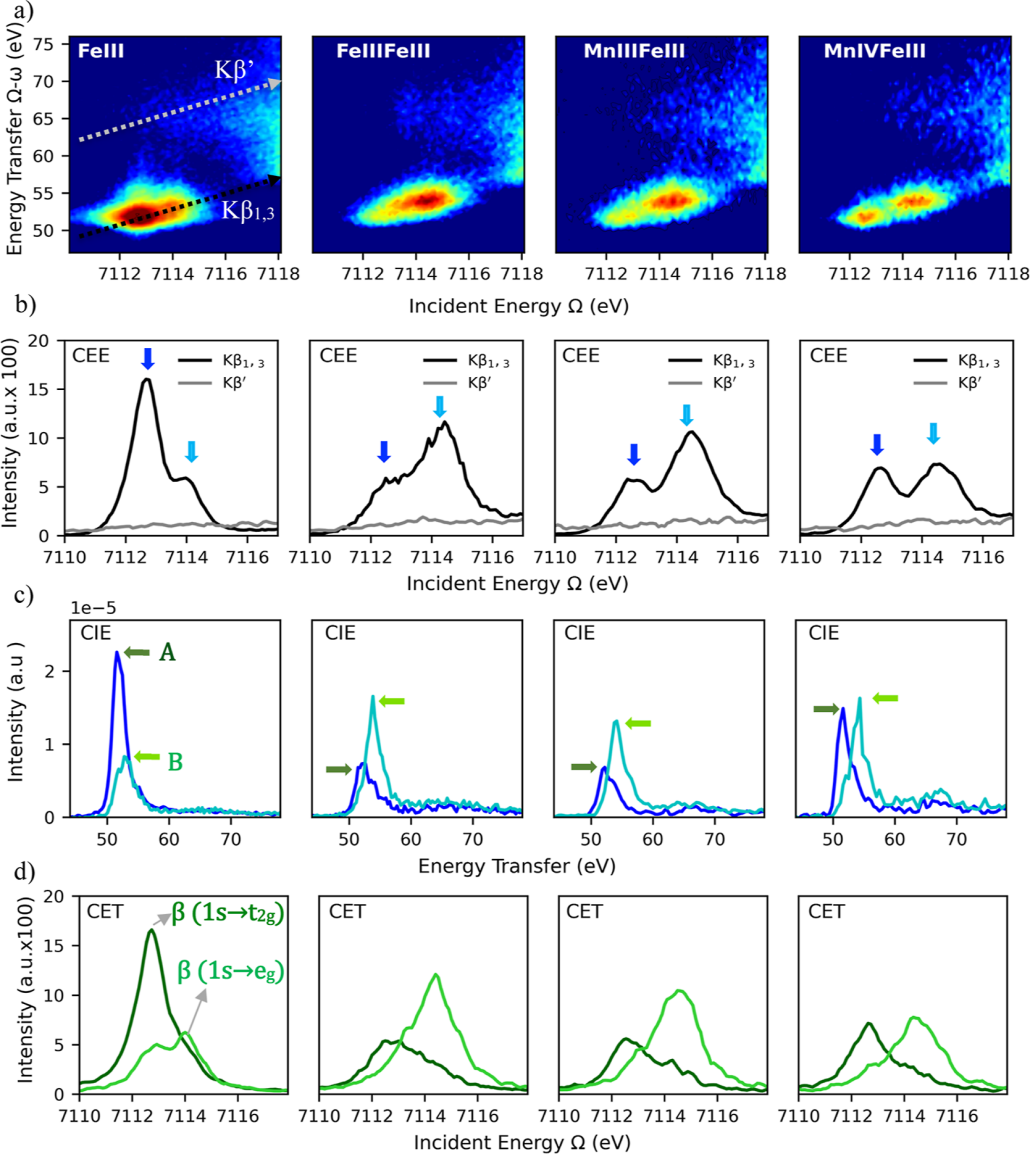
(a) Fe 1s3p RIXS maps of the pre-edge region for FeIII, FeIIIFeIII, MnIIIFeIII, and MnIVFeIII. (b) CEE cuts along the α-(Kβ′) and β-(Kβ_1,3_) channels (as shown in the FeIII RIXS plane of panel a). (c) CIE cuts taken at the positions indicated by the blue and cyan arrows in panel b. (d) CET cuts taken at the constant incident energies indicated by the arrows A and B in panel (c).

The Fe 1s3p RIXS planes for all models are shown in Figure 6a along with the corresponding CEE (b), CIE (c), and CET (d) cuts. All planes contain a pre-edge feature with an asymmetric intensity distribution indicating contributions from multiple excitations, which are best analyzed by looking at cuts of the plane. For clarity, the location of the diagonal cuts for both α-(Kβ′) and β-(Kβ_1,3_) channels are illustrated in the FeIII RIXS plane. For all of the Fe 1s3p RIXS planes, the pre-edge features are closely aligned with the Kβ_1,3_ diagonal.

Figure 6b shows the CEE cuts in the pre-edge region, with the Kβ_1,3_ (black) and Kβ′ HERFD-XAS (gray) cuts corresponding to the β-channel and the α-channel, respectively. The Kβ′ CEE plots show no intensity for any of the iron models, while Kβ_1,3_ shows two main features. The lack of intensity in Kβ′ CEE plots indicates that there is no available α-channel in a d^5^ high-spin configuration. Importantly, these data highlight the ability of this method to assess the local spin state of a transition metal via the separation of α- and β-excitation channels.

For Figure 6c, a CIE cut was selected by utilizing the maxima of each Fe pre-edge feature (as indicated by the arrows in Figure 6b). The selectivity of a constant incident energy cut will depend on the relative energetic separation of the selected intermediates and also the bandwidth of the incident X-ray beam. The resulting resonant XES (or CIE) spectra (here plotted on the energy transfer axis) correspond to distinct sets of final states (3p^5^3d^*n*+1^). Despite being M-edge-like final states, we note however that the selection rules differ between 1sp3p RIXS M-edge-like final states and true M-edge XAS, which will be discussed further in the analysis below.

Importantly, unlike M-edge XAS, the CIE cuts of the 1s3p RIXS planes allow for the separation of a range of final states resulting from an electron occupying either the 3d-π or the 3d-σ set of MOs. The resulting M-edge-like final states can thus in principle be separated into the β π (t_2g_) (blue) and σ (e_g_) (cyan) contributions (Figure 6c). Here, it is, however, important to acknowledge that the selection rules are modulated relative to the M-edge in everything except rigorously centrosymmetric complexes. In a direct M-edge process, dipole-allowed 3p to 3d transitions dominate the intensity mechanism, whereas in the indirect RIXS process, the initial 1s to 3d transition is dipole forbidden and the states with 3d-4p mixing dominate the intensity mechanism. Hence, as the symmetry is lowered from a pure *O*_*h*_ limit (where p-d mixing is forbidden) to the more locally *C*_4*v*_-like limit in the dimeric oxo complexes, the 3d-4p mixing will dominate the spectral intensity of the CIE cuts. For the oxo complexes, the primary mechanism for p-d mixing is the short metal-oxo bond, which enables d_*z*^2^_-p*z* through covalent mixing.

Hence, for FeIIIFeIII, MnIIIFeIII, and MnIVFeIII, the CIE cuts effectively map the oxo bridge groups’ contribution to the spectra, which is covalently mediated by 3d/4p mixing. The dark blue CIE peak corresponds to the π-contribution, and the cyan CIE peak corresponds to the σ-contribution. By comparing the CEE cuts in panel b with the CIE cuts in panel c, one readily observes that the CIE cuts provide an alternative means to deconvolute the contributions to the pre-edge, albeit with reduced resolution compared to the CEE cuts. For direct overlays of the CEE and CIE cuts, see Figure S2. Due to the limited resolution in the CIE direction (see Table S4), we refrain from further quantitative analysis. We note, however, that the CIE cuts are needed to determine the energies for the CET cuts.

Figure 6d shows the CET plots with constant energy transfer values matching the maxima of each CIE cut (labeled as “A” and “B” in Figure 6c). All models show that by selecting a different constant energy transfer, the pre-edge region is deconvoluted into two main features. These features are the consequence of selecting specific CFSs that can only be accessed by intermediates generated via 1s → 3d-t_2g_ (dark green, CET “A”) and 1s → 3d-e_g_ (light green, CET “B”) transitions. The energy splitting for CET follows a similar trend as the CEE cuts: ∼1.3 eV for FeIII, ∼1.7 eV for FeIIIFeIII, ∼1.95 eV for FeIIIMnIII, and ∼1.8 eV for FeIIIMnIV. Figure S3 overlays CEE and CET cuts, showing an overall overlap between features with minor differences in the splitting, as they both originate from the same final states.

**Figure 7:**
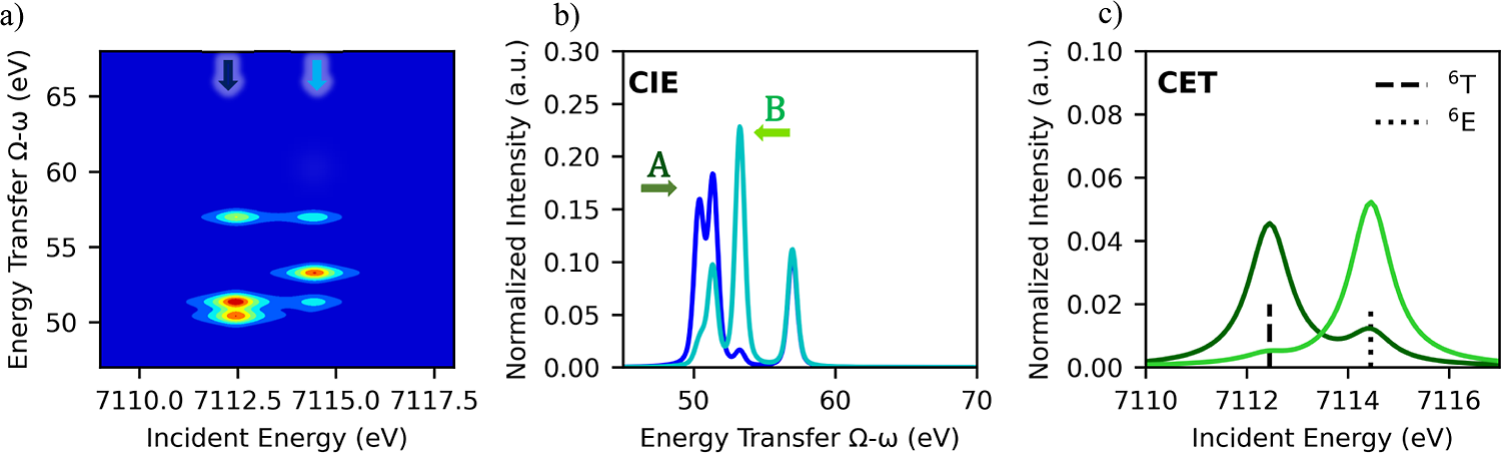
Simulated 1s3p RIXS for the *O*_*h*_ limit with 10Dq = 2 eV Fe(III) (a) and the corresponding CIE (b) and CET (c) slices of the plane. The dark and light blue arrows in (a) correspond to the fixed incident energies chosen to plot the CIE curves in (b), color coded. The A and B features in (b) correspond to the energy transfer values chosen for the CET slices in (c), respectively. Dashed and dotted lines in (c) indicate intermediate state multiplets populated at specific incident energies.

**Figure 8:**
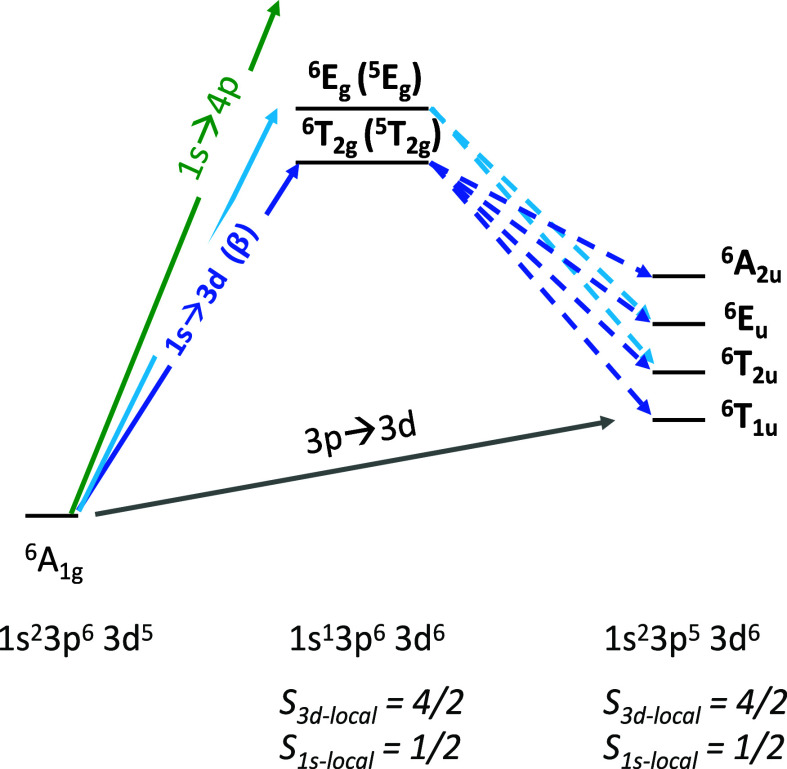
Simplified multiplet diagram scheme for the Fe(III) 1s3p RIXS processes. Intermediate states include the full state term symbol and the 3d^*n*+1^-local state term symbol in parentheses.

The Fe 1s3p RIXS process was also investigated computationally to gain a full understanding of the information contained in the experimental 2D RIXS map and the sensitivity of the technique to changes in the metal electronic structure. Figure 7 shows the simulation of the 1s3p RIXS plane for an Fe(III) atom in the *O*_*h*_ ligand field (10Dq = 2). When slicing the plane, both the calculated CIE (7b) and CET (7c) spectral cuts parallel the general experimental trends. The nature of the states involved in the 1s3p RIXS process can be understood by evaluating the total multiplicities of the initial, intermediate, and final states, and their local (i.e., within the 3d level only) multiplicities. To address this in a pedagogical way, Figure 8 displays a multiplet diagram of the RIXS process, including the total and local spin multiplicities. It is important to note that this is a simplified diagram, including only the dominant transitions. Note that the local spin state refers to the 3d shell, and it is dictated by the spin channel. Thus, all excited and final states reached via the same spin channel (for the same metal oxidation state) will share the same 3d local multiplicity.

In the Fe(III) 1s3p RIXS process, excitations from the 1s into unoccupied 3d levels give rise to 1s^1^3p^6^3d^5+1^ and 1s^2^3p^5^3d^5+1^ intermediate and final states, respectively. These states must also account for conservation of spin in 1s3p RIXS due to the lack of a 1s spin orbital and weak 3d and 3p spin orbit coupling. As explained in the XAS Pre-Edges section, in the high-spin ferric case, the 1s-3d resonant excitations are only possible on the β-excitation channel (Figures 4c and 8) and result in a local spin of 4/2 (*S* = 2) for the d^5+1^ configuration or an overall sextet when including the unpaired α electron created in the 1s shell (*S*_(1s-local)_ = 1/2). Once the 1s(β) hole is filled by a 3p(β) electron during the emission process, the resulting Fe(III) final state conserves the same total spin, and thus it will be primarily dominated by sextets ^6^Γ [2*S*_total_ + 1 = 2(4/2_(3d-local)_ + 1/2_(3p-local)_) + 1], which are locally (in the 3d level) quintets. These sextet final states are grouped in the energy window ∼49–56 eV (energy transfer axis). The energy shift between the maxima of these CIE plots reflects the further deconvolution of the different ^6^Γ states when considering the symmetry, as the blue and cyan cuts are due to β excitations to the 3d_t_2g__ and 3d_e_g__-like set, respectively. A further analysis is provided by relying on the simplified Fe(III) multiplet diagram (Figure 8) and simulated results (Figure 7).

As shown in Figure 8, for high-spin Fe(III), the ^6^Γ ground state (terms are determined by the arrangement of electrons in the 3d shell) transforms as ^6^A_1g_ in *O*_*h*_ symmetry, and the electric quadrupole and dipole operators transform at T_2g_/E_g_ and T_1u_, respectively. Upon excitation into the d-manifold [⊗(T_2g_ ⊕ E_g_)], the resulting ^6^Γ excited states are locally 3d^*n*+1^ quintet states ^5^T_2g_ and ^5^E_g_. Each of these intermediate states will give rise to spectral intensity in specific final states during the emission process, which can be determined by taking the direct product with the dipole operator T_1u_ [⊗(T_1u_)]. The 1s^2^3p^5^3d^6^ final state configuration gives rise to 4 terms in Oh that contribute to the pre-edge 1s3p RIXS spectrum: ^6^A_2u_, ^6^E_u_, ^6^T_1_, and ^6^T_2u_. These final states can be identified computationally and grouped by the origin of their excited states in the CIE plots (Figure 7b), where the blue and cyan curves correspond to the ^6^T_2g_ and ^6^E_g_ states, respectively. The energy splitting of these features thus reflects the energy difference between the local (3d level) ^5^T_2g_ and ^5^E_g_ excited states, while the intensities reflect the differences in symmetry allowed mixing for the states with π (T) and σ (E) character. We note that in a LF multiplet model, as presented here, anisotropic covalency is not accounted for.

Simulated CET is also shown in Figure 7c and shows good agreement with the experimental results. As mentioned in the experimental part, with the use of CET cuts, it is possible to individually identify the ^6^T_2g_ (3d local ^5^T_2g_) and ^6^E_g_ (3d-local ^5^E_g_) excited state contributions to the pre-edge region; by scanning over a 10Dq range, one can monitor how the ligand field affects each individual excited state. The CET cuts thus could be evaluated in analogy to the d^6^ Tanabe Sugano diagram when only considering the local terms.

### Mn 1s3p RIXS

**Figure 9:**
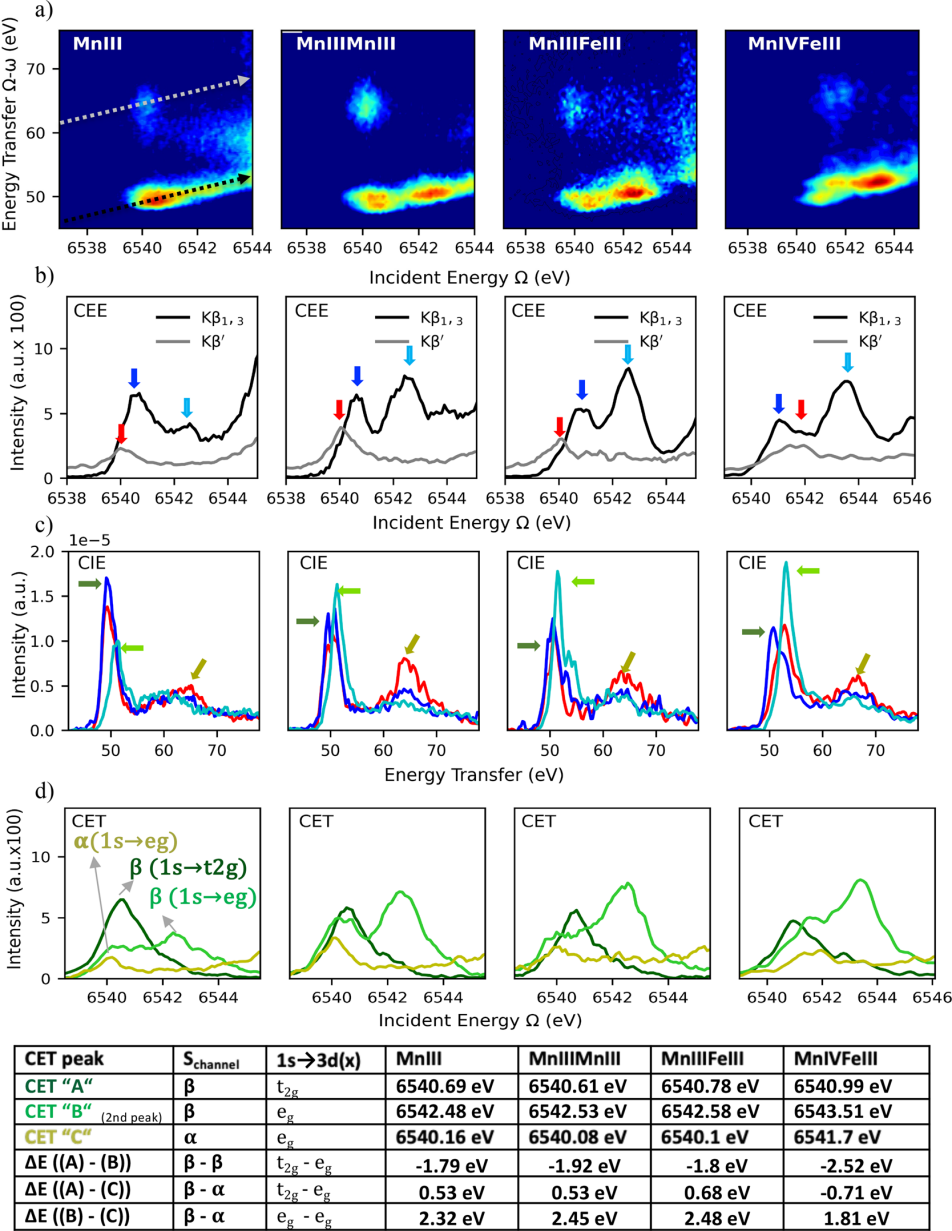
Top: Mn 1s3p RIXS planes of the pre-edge region of MnIII, MnIIIMnIII, MnIIIFeIII, and MnIVFeIII (a) and corresponding CEE (b), CIE (c), and CET (d) cuts. Bottom: summary of the CET peaks, including the spin channel and transition involved, the energy position of the peak maxima, and the energy difference between these peaks.

The Mn 1s3p RIXS planes for all models are shown in Figure 9a together with the corresponding CEE (b), CIE (c), and CET (d) slices of each plane. For the interpretation of the spectra below, we focus on the d-electron configuration on each Mn atom and consider all Mn models to have their unpaired electrons with a local spin up configuration (α), including the antiferromagnetic MnIIIFeIII and MnIVFeIII models.

**Figure 10:**
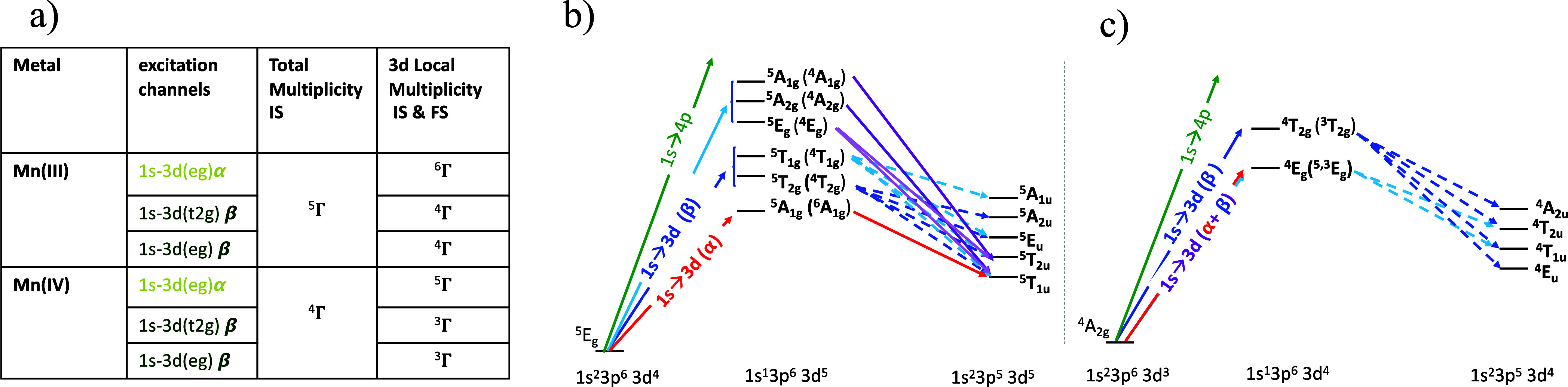
(a) Multiplet table and simplified diagram scheme for Mn(III) (b) and Mn(IV) (c) 1s3p RIXS processes. Intermediate state process includes the total spin state and the 3d^*n*+1^-local spin state in parentheses.

Figure 9a shows that all of the Mn RIXS planes contain two different groupings of transitions on the energy transfer axis. One at ∼50 eV and the second at ∼65 eV aligned within the Kβ_1,3_ (β-channel) and Kβ′ (α-channel) diagonals, respectively. The presence of the ∼65 eV feature, which was absent in the Fe 1s3p RIXS planes, can be easily rationalized due to the fact that in high-spin Mn(III) (d^4^) and Mn(IV) (d^3^) α-excitation channels are possible. As in the Fe case, the Kβ_1,3_ region is somewhat asymmetric and seems to contain two features, whose intensity distribution and energy separation vary across the series. These changes in intensity and energetic splitting can be further analyzed by slicing the plane. The CEE plots in Figure 9b of the pre-edge features along the Kβ_1,3_ and Kβ′ diagonals contain the β- and α-excitation channels, respectively. These slices of the plane allow the excited states to be separated by their local 3d multiplicity. A summary of the possible spin channel Mn 1s3p RIXS processes is presented in Figure 10a. Figure 9b shows the CEE cuts at the Kβ′ energy (HERFD) for all models (gray lines). In contrast to the Fe case, these CEE cuts exhibit a clear pre-edge feature. This is due to available 1s → 3d-e_g_(α) transitions for all models, giving ^6^Γ [Mn(III) case] and ^5^Γ [Mn(IV) case] 3d intermediate states, as shown in Figure 10a. For the Mn(III) cases, the Kβ′ CEE pre-edge is found at lower energies than the Kβ_1,3_ pre-edge, while for the Mn(IV) case, the pre-edge peak in Kβ′ CEE is found at higher energies than the low-energy peak in Kβ_1,3_ CEE. This is consistent with the local *S* = 5/2 3d configuration [reached upon a 1s to 3d excitation in the Mn(III)] having a greater exchange stabilization than the local *S* = 4/2 3d configuration reached in the Mn(IV) case.

Figure 9c shows the CIE cuts of the Mn 1s3p RIXS plane for all models. Constant incident energies were selected to match the maxima for each Mn pre-edge feature in both CEE cuts. The corresponding M-edge like-final states for the Mn case can be deconvoluted into β–π (t_2g_) (blue), β–σ (e_g_) (cyan), and α–σ (e_g_) (red) contributions (Figure 9c). As was the case for the Fe 1s3p RIXS planes discussed above, the CIE cuts effectively provide a means to identify the major envelopes of transitions contributing to the pre-edge region, albeit at somewhat more limited resolution (Figure S4 and Table S4). Hence, the primary reason for showing these data is to establish the energies for the CET cuts, which we discuss next.

Figure 9d shows the CET obtained by selecting constant energy transfer values corresponding to the energy positions in Figure 9c labeled as “A,” “B,” and “C”. As in the Fe case, the CET can be aligned with the pre-edge features of the CEE (or HERFD) cuts (Figure S5). Figure 9 (bottom) summarizes the energy positions of the features contained in each CET cut and the energy difference between them. Starting with the lowest constant energy transfer in Figure 9c, CET “A” slices the plane at ∼49 eV for Mn(III) cases and ∼51 eV for the Mn(IV) case. These cuts correspond mainly to final states reached via 1s → 3d_t_2g__-like set (β) excitation and, in all cases, align with the first pre-edge feature of the Kβ_1,3_ CEE plot in Figure 9b. CET “B” cuts (light green) are distributed into two features, located at ∼6540–6541 eV (1st peak) and ∼6542–6543 eV (2nd peak). In an orbital-based picture, the excited states contained in CET “B” cuts are expected to align with the 1s → 3d_e_g__-like set (β) excitation in Kβ_1,3_ pre-edge HERFD (Kβ_1,3_ CEE, Figure 9b). However, clear pre-edge features at lower energy that align with the α 1s→ 3d_e_g__-like set are observed in all cases. This can be readily understood from simple pen and paper calculations of the multiplets involved. A simplified multiplet diagram of both Mn(III) and Mn(IV) RIXS is shown in Figure 10b and c, respectively. In the Mn(III) case, the α and β transitions to the e_g_-like set give rise to local ^6^A_1g_ and ^4^A_1g_ intermediate states, respectively (Figure 10b). However, as both intermediate states are overall ^5^A_1g_ states, both contributions are seen here. Analogously, in the Mn(IV) case, both the α and β transitions to the e_g_-like set give rise to local ^5^E_g_ and ^3^E_g_ intermediate states, respectively, which are overall of ^4^E_g_ total symmetry and thus also contribute. To simplify the Mn(IV) multiplet diagram, both α and β transitions giving rise to ^4^E terms are grouped under the same 1s-3d transition in Figure 10c. A more complete picture of the intermediate states for both Mn(III) and Mn(IV) is given in the Supporting Information where all pre-edge states are tabulated (Tables S5 and S6), and simulations of the pre-edge XAS in Figures S6 and S7 show which states give intensity. Finally, we note that an “isolated” α 1s→ 3d_e_g__-like set is seen in the CET “C” cut (Figure 9d, gold line), providing further experimental support for this assignment. It is of interest to note that the difference in energy between the CET “C” and CET “B” cuts (bottom line of the table in Figure 9) is larger for Mn(III) than it is for Mn(IV). This is attributed in part to the greater exchange stabilization of the local sextet state, which is reached in the Mn(III) case relative to the local quintet, which is reached in the Mn(IV) case.

**Figure 11:**
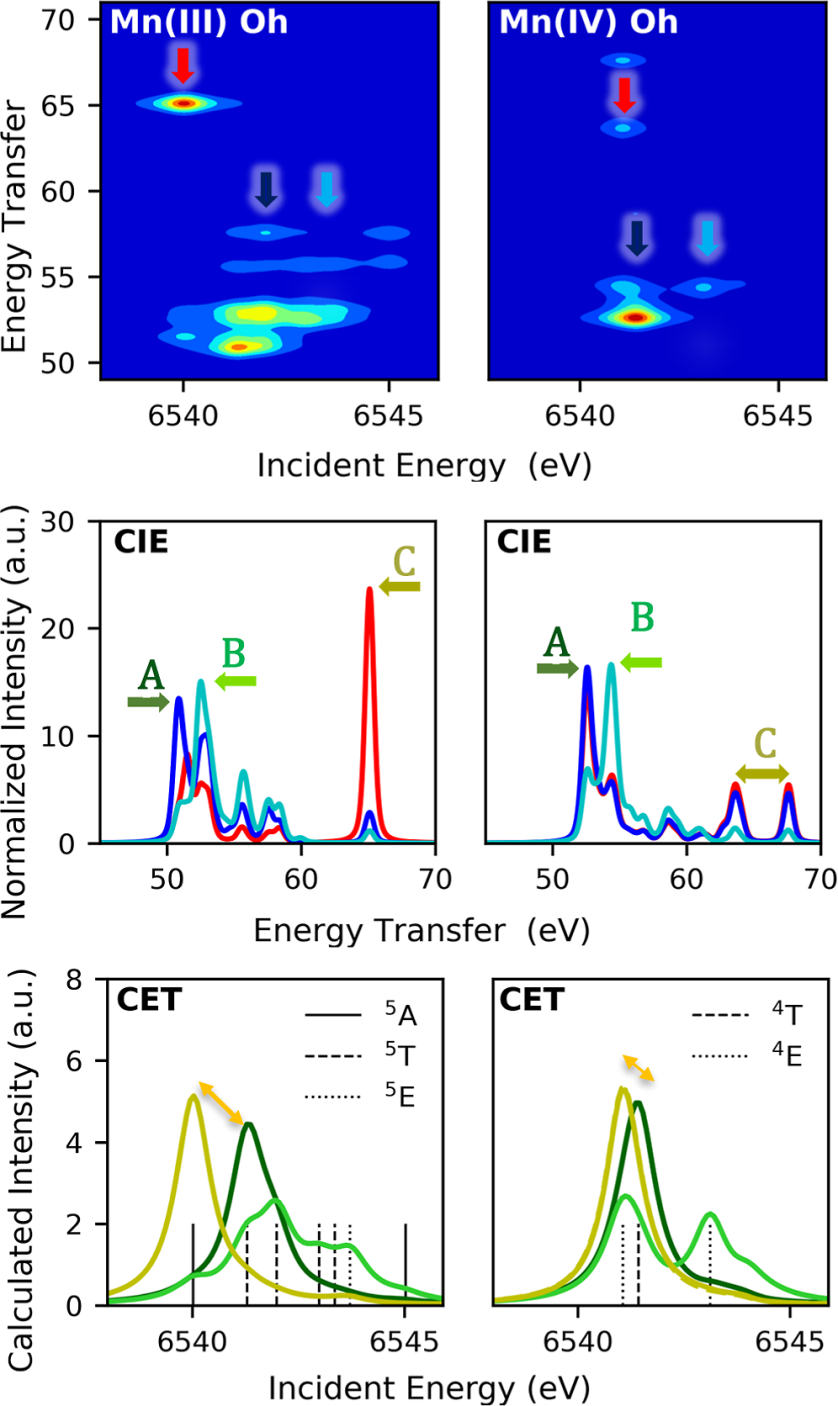
Calculated 1s3p RIXS for the *O*_*h*_ limit with 10Dq = 2 eV Mn(III) (left) and Mn(IV) (right) and the corresponding CIE and CET slides of the plane. The orange arrow in the CET plots corresponds to the Δ*E* (A–C). Only states providing 80% of intensity in CET are printed.

Similar to the Fe case, Figure 11 shows the simulated RIXS planes, CIE, and CET cuts for *O*_*h*_ Mn(III) (left panel) and Mn(IV)(right panel). The planes are in good qualitative agreement with the experimental results shown in Figure 9a. A further discussion of these calculations and the effects of Jahn–Teller distortions in the Mn(III) case can be found in Figures S8 and S9. A mismatch is found in the energy position of CET 3d_t_2g__(β) relative to those of the other intermediates. Figures S10 and S11 prove that this disagreement is ligand field-dependent and can also be further corrected by reducing the Salter–Condon parameters to account for high covalency. Importantly, these multiplet calculations help support the simple pen and paper calculations that were utilized in the preceding analysis.

### Effect of the Heterometal Centers in CFS Spectra

**Figure 12:**
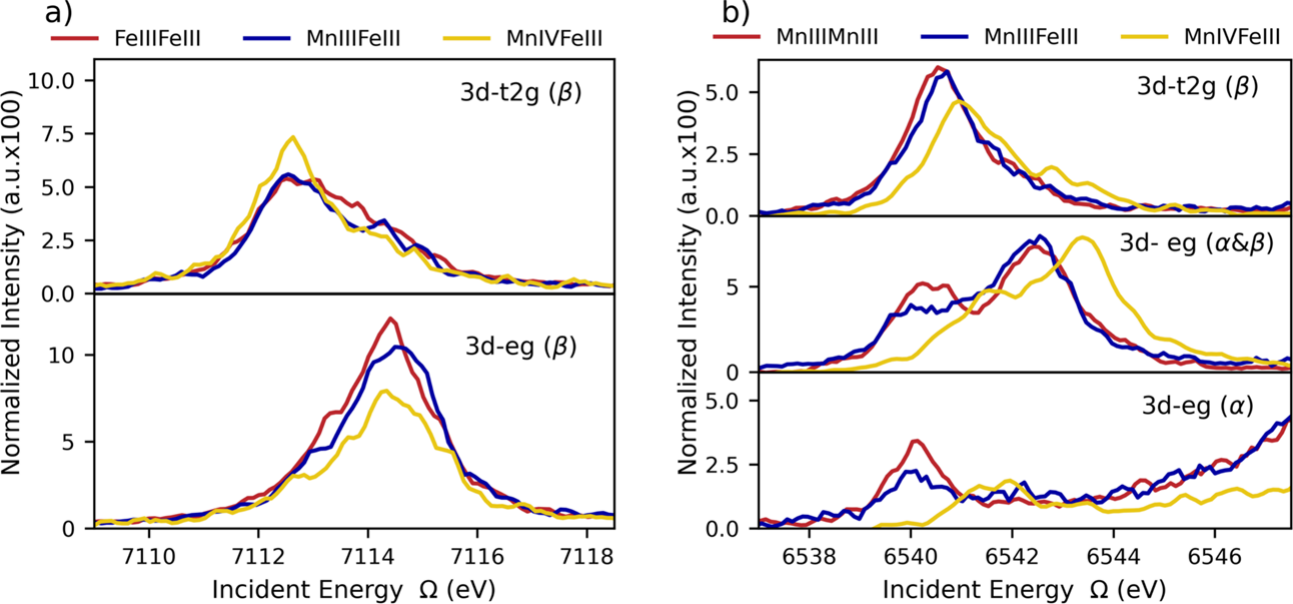
Fe (a) and Mn (b) overlap of experimental CET with excited states reached via populating Fe β 3d-t_2g_ and e_g_ levels (left) and Mn α and β 3d-t_2g_ and e_g_ levels (right) for FeIIIFeIII (pink), MnIIIMnIII (pink), MnIIIFeIII (blue), and MnIVFeIII (olive) models.

In the previous sections, we analyzed the Fe and Mn 1s3p RIXS individually and have provided a detailed analysis of the information content of the RIXS planes. In this section, we now evaluate the perturbations that occur in the Fe and Mn CFS spectra ongoing from the homometallic to the heterometallic systems in tandem. We begin by evaluating the overlaid Fe CET cuts for FeIIIFeIII, MnIIIFeIII, and MnIVFeIII (Figure 12a, top panel) for the 3d-t_2g_ and 3d-e_g_ β final states. These plots clearly show that the presence of Mn modulates the electronic structure of the Fe site relative to that of the homodimer. Of particular interest is the variation in the states corresponding to occupation of the 3d-t_2g_ β orbitals, as this is the lowest energy π-interacting LUMO. Here, one observes that the presence of Mn lowers the intensity-weighted average energy of 3d-t_2g_ β intermediate state envelope in both heterodimers relative to the homodimer (on the order of 0.4 eV). Upon inspection of the analogous Mn CET cuts (Figure 12b, top panel), one observes a reverse trend with the energy of the 3d-t_2g_ β state cuts shifting to higher energy ongoing from the homodimeric MnIIIMnIII to heterodimeric MnIIIFeIII (in this case by ∼0.2 eV) and a larger shift (of 0.6 eV), as may be expected, for the MnIVFeIII. Although the shifts between the trivalent homo- and heterodimers may appear relatively small, it is important to keep in mind that 0.2 eV corresponds to ∼5 kcal/mol. If one relates this state-based picture to the frontier molecular orbitals involved in chemical reactivity, this is certainly a significant enough energy difference to modulate reaction pathways in catalytic systems.

In the context of reactivity, it is also of interest to examine the energetics of the Mn 3d-e_g_ α final state cuts (Figure 12b, bottom panel), which for a catalytic system would relate to the energetics of a σ-based electron transfer process. Ongoing from the MnIIIFeIII to MnIVFeIII models, the energetic separation between the lowest lying Mn 1s to α-3d (Figure 12b, bottom panel) and β-3d transitions (Figure 12b, middle panel) has not only decreased but in fact the energy ordering inverts, with the energetically lowest resonant state 1s^1^3d^*n*+1^ corresponding to populating the Mn 3d β LUMO (^5^Γ), at −0.7 eV (or ∼−16 kcal/mol) from the Mn 3d α LUMO (^3^Γ), in the MnIV case.

This is in contrast to the MnIII case, where the lowest energy transition results from populating the α-LUMO. The comparison of the CET final state cuts also highlights the importance of selectively examining the energies of specific final states.

## Discussion

In this study, we have shown that a far more detailed and quantitative description of the transition metal electronic structure can be obtained through the analysis of experimental and calculated 1s3p RIXS planes.

CEE cuts along the Kβ′ and Kβ_1,3_ emission energies allow, respectively, the α and β excitation channels within the pre-edge region to be separately identified and quantitatively evaluated. CIE cuts allow one to obtain M-edge like final states and provide a means to deconvolute the π (t_2g_) and σ (e_g_) contributions to the pre-edge, albeit at a reduced resolution relative to the CEE cuts. CET slices of the plane allow for the separation of the individual excited states contributing to the pre-edge region. Specifically, we compared the information provided by CET and CEE. The advantage of CET cuts lies in their ability to directly and experimentally deconvolute these contributions, avoiding the ambiguities of peak assignments via fitted deconvolutions. CET cuts enable the sorting of pre-edge features according to their contributing intermediate state terms, thereby offering an experimental method to assess the energetics of the contributing multiplets. In our view, the latter is thus a particularly valuable finding for evaluating subtle electronic structural changes and how they may correlate with reactivity, as ultimately, it is the energy of these states that dictate the accessible chemical transformations.

In this context, we have shown in the previous section that, ongoing from the homometallic to the heterometallic complexes, subtle modulations occur in both the energetics of the T_2g_ and E_g_ final states as well as in the covalency of each metal site. Importantly, we also illustrate that in cases where there are doubly unoccupied d-orbitals, it is possible to distinctly observe the α and β contributions to the final states, and hence to probe 3d local multiplets with different spins. The shifts in the energies of the intermediate states in the homo vs heterodimers are observed to be on the order of ∼5–14 kcal/mol and thus indicate a significant electronic structural modulation. In the case of catalytic or enzymatic systems, such changes would certainly be enough to strongly modulate favored reaction pathways. These modulations (which are not observed in the XAS or XES alone) highlight the power of 1s3p RIXS to provide more detailed electronic structural information and provide hints as to why certain biological active sites may only achieve optimal reactivity with heterometallic active sites.

In our view, 1s3p RIXS can be particularly impactful in catalysis research, as it may provide a means to experimentally evaluate the importance of two-state/multistate reactivity that has been proposed to be of particular importance for the reactivity of high-valent iron and manganese catalysts.^103−105^ In this context, we highlight a recent 2p3d RIXS study, which was utilized to experimentally determine the triplet to quintet energy gaps in a series of Fe(IV)-oxo complexes and make direct correlations to two-state reactivity.^106^ We note that a recent 1s2p RIXS study by Braun et al.^107^ has suggested that the assignment of the final state multiplets in Fe(IV)-oxo complexes will vary depending on the utilized theoretical interpretation tool, which they suggest is exacerbated by TDDFT underestimating α/β spin polarization. The experiments presented herein have the advantage of being able to directly experimentally identify the spin multiplicity of the final states, thus bypassing the potential limitations of the utilized theoretical approach and providing an experimental means to address open questions in the field.

In the context of this study, the MnIVFeIII complex is of particular interest, as it has the same valency as the key active intermediate in class Ic RNRs. In RNR, the MnFe cofactor is responsible for radical initiation, directly abstracting a H atom from tyrosine, the detailed mechanism of which is a subject of ongoing research.^108^ In the broader field of C–H bond activation, H atom abstraction by high-valent transition metal oxo complexes has been extensively discussed. In particular, C–H activation by *S* = 2 Fe(IV)-oxo complexes has been shown to proceed via σ-based pathway, as they reach similar high exchange stabilization to that discussed herein for the 3d local ^6^Γ in Mn(III) cases. For *S* = 1 Fe(IV)-oxo complexes, it is suggested that reactivity can occur on either a π-based pathway, making use of the unoccupied β t_2g_ spin orbitals or a σ-based pathway, making use of the unoccupied low-lying α-based d_*z*^2^_ orbital. In contrast, two-state reactivity in C–H activation by Mn(IV)-oxo complexes^104,109^ in *D*_4*h*_ symmetry is thought to involve promotion of the ^4^B_1_ ground state to a ^4^E state by transferring an electron from e (d_*xz*_, d_*yz*_) to b_1_ (d_*x*^2^–*y*^2^_) during the hydrogen-atom-transfer process. In the presence of a substrate, the transfer of an β electron from the C–H σ-bonding orbital to the β-LUMO ^4^B ground state can give rise to a local ^3^Γ state. However, electron transfer to the ^4^E state can lead to local ^5^Γ and ^3^Γ states. It is important to note the different nomenclature used in the literature in terms of reactivity, where the σ-pathway is related to the Mn–O donation, while in this study, we simply refer to the 3d sigma set of orbitals (3d-e_g_). Nevertheless, the states which are invoked in tuning reactivity are those which we access in a 1s3p RIXS experiment. By directly probing the energetics of the low-lying excited states that are involved in π- vs σ-reaction pathways, 1s3p RIXS provides a means to experimentally test important concepts in two-state reactivity. We thus envision that 1s3p RIXS has the potential for broad impact on many areas of catalysis research. Furthermore, the detailed electronic structural information provided by 1s3p RIXS may have an equal impact in other areas—ranging from magnetism to materials chemistry.

## Data Availability

Experimental and simulated data supporting the conclusions of this work are available in the Edmond open data repository of Max Planck Society at doi:10.17617/3.B8I2LJ.

## Supporting Information Available

Additional experimental details and computational details, including tabulated pre-edge energy positions and emission energies splitting, further comparisons between different slices of the RIXS planes for all models, and further computational analysis for all models varying ligand field parameters (PDF)
