# Supplementary material for: Experimentally Assessing the Electronic Structure and Spin-State Energetics in MnFe Dimers Using 1s3p Resonant Inelastic X-ray Scattering
Source: Inorg Chem. 2024 Sep 16;63(39):18468–83. doi: 10.1021/acs.inorgchem.4c01538 (PMC11445731; doi:10.1021/acs.inorgchem.4c01538)
Supplement: Supplementary file 1 — ic4c01538_si_001.pdf [file ic4c01538_si_001.pdf]

SUPPORTING INFORMATION

Experimentally assessing the electronic structure and spin state energetics in MnFe dimers using 1s3p resonant inelastic X-ray scattering

Rebeca G. Castillo,[a,b] Benjamin E. Van Kuiken,[c] Thomas Weyhermüller,[a] and Serena DeBeer [a*]

[a] Max Planck Institute for Chemical Energy Conversion, Stiftstrasse 34, D-45470 Mülheim an der Ruhr, Germany

[b] Laboratory of Ultrafast Spectroscopy (LSU) and Lausanne Centre for Ultrafast Science, École Polytechnique Fédérale de Lausanne (EPFL), CH-1015 Lausanne, Switzerland

[c] European XFEL, Holzkoppel 4, D-22869 Schenefeld, Germany

*Correspondence to: serena.debeer@cec.mpg.de

Table of Contents

Table S1. Splitting between the $K\beta_{1,3}$ and Kβ'XES features for the non-resonant Fe and Mn Kβ XES spectra S3

Table S2. Atomic terms for non-resonant Kβ XES and 1s3p RIXS S3

Table S3. Tabulated Pre-edge energy positions and IWAEs S3

Figure S1. Integrated area for both Fe and Mn CIE A and B peaks for all model complexes and DFT analysis S4

Figure S2. Overlays of Fe CIE vs CEE S5

Figure S3. Overlays of Fe CEE vs CET S5

Figure S4. Overlays of Mn CIE vs CEE S5

Figure S5. Overlays of Mn CEE vs CET S6

Table S4. 1s3p RIXS instrumental and lifetime broadenings for Fe and Mn 1s3p RIXS S6

Figure S6. Pre-edge XAS Simulation of Mn(III) S7

Table S5. Mn(III) core-excited states S7

Figure S7. Pre-edge XAS Simulation of Mn(III) S8

Table S6. Mn(III) core-excited states S8

Figure S8. MnII Oh and Jahn Teller energy levels S9

Figure S9. Mn(III) simulated RIXS map for O_h and D_{4h} S10

Figure S10. Calculated CET for MnIV varying 10Dq S11

Figure S11. Effects of scaling Slater-Condon Parameters in Mn(IV) S12

REFERENCES S12

Table S1. Splitting between the $K\beta_{1,3}$ and Kβ' XES features for the non-resonant Fe and Mn

**Kβ XES**				**1s3p RIXS**			
**$3d^n$**	GS ($1s^2 3d^n$)	IS* ($1s^1 3d^n$)	FS ($1s^2 3p^5 3d^n$)	$3d^n$	GS ($1s^2 3d^n$)	IS ($1s^1 3d^{n+1}$)	FS ($1s^2 3p^5 3d^{n+1}$)
**d^3**	^4F	$^{3,5}F$	$^{3,5}D$, $^{3,5}F$, $^{3,5}G$	d^3	^4F	^4D	^4P, ^4D, ^4F
**d^4**	^5D	$^{4,6}D$	$^{4,6}P$, $^{4,6}D$, $^{4,6}F$	d^4	^5D	^5S	^5P
**d^5**	^6S	$^{5,7}S$	$^{5,7}P$	d^5	^6S	^6D	^6P, ^6D, ^6F
**d^6**	^5D	$^{4,6}D$	$^{4,6}P$, $^{4,6}D$, $^{4,6}F$	d^6	^5D	^5F	^5D, ^5F, ^5G
**d^7**	^4F	$^{3,5}F$	$^{3,5}D$, $^{3,5}F$, $^{3,5}G$				

Kβ XES spectra.

Table S2. Atomic terms for or non-resonant Kβ XES and 1s3p RIXS .

**Complex**	**ΔE ($K\beta_{1,3}$ - Kβ')**	
	**Fe**	**Mn**
FeIII	14.2 eV	-
MnIII	-	15.7 eV
FeIIIFeIII	14.5 eV	-
MnIIIMnIII	-	15.6 eV
MnIIIFeIII	14.1 eV	15.3 eV
MnIVFeIII	14.2 eV	13.9 eV

Table S3. Fe and Mn pre-edge peaks energy positions from voight fits (Peak1 and Peak2), energy splitting (ΔE) and intensity-weighted average pre-edge energies (IWAEs)

**Fe $K\beta_{1,3}$ HERFD pre-edge**	**Peak1 (eV)**	**Peak 2 (eV)**	**ΔE (eV)**	**IWAEs (eV)**
FeIII	7112.72	7114.14	1.42	7113.13
FeIIIFeIII	7112.63	7114.43	1.80	7114.04
MnIIIFeIII	7112.45	7114.51	2.06	7114.11
MnIVFeIII	7112.56	7114.54	1.98	7113.98
**Mn $K\beta_{1,3}$ HERFD pre-edge**	**Peak1 (eV)**	**Peak 2(eV)**	**ΔE (eV)**	**IWAEs (eV)**
MnIII	6540.69	6542.39	1.70	6541.60
MnIIIMnIII	6540.70	6542.62	1.92	6542.05
MnIIIFeIII	6540.80	6542.57	1.77	6541.94
MnIVFeIII	6541.05	6543.52	2.47	6543.57

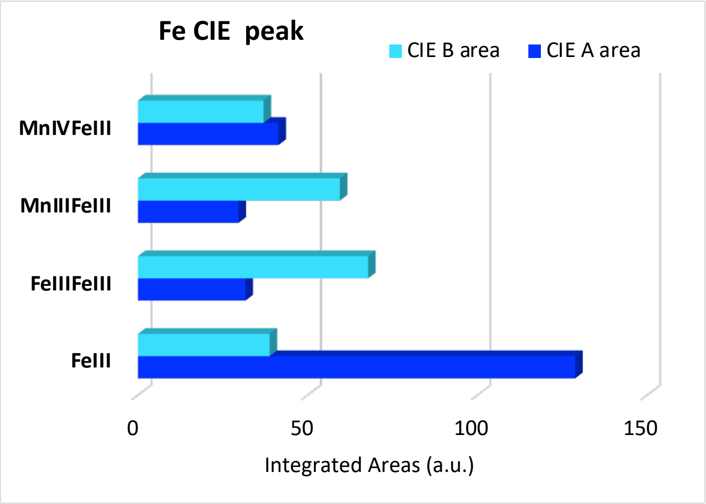

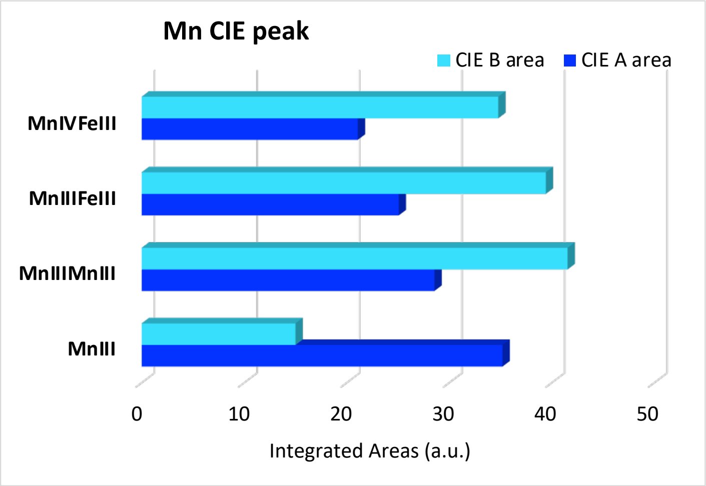

Figure S1. Integrated area for both Fe and Mn CIE A and B peaks for all model complexes.

Areas are determined by numerical integration along the energy range comprising the main CIE peak.

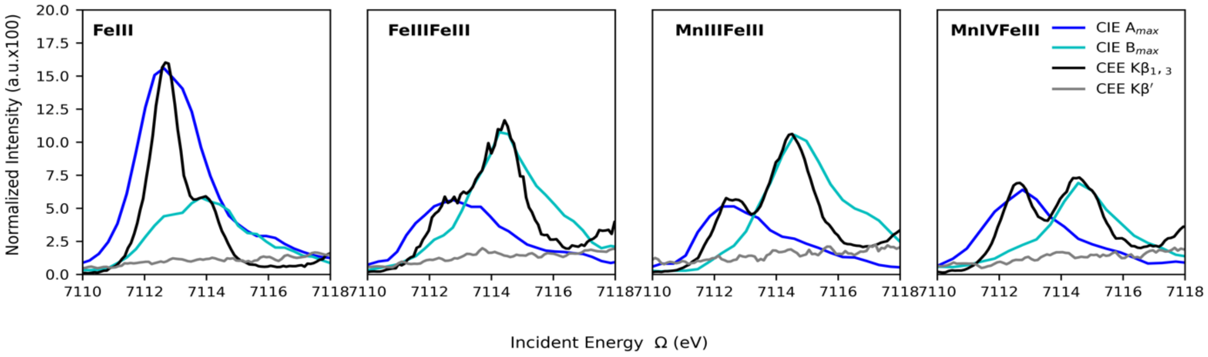

Figure S2. Overlays of Fe CIE vs CEE within the pre-edge region along the incident energy axis. CIE data was shifted so CIE A matches the chosen constant incident energy.

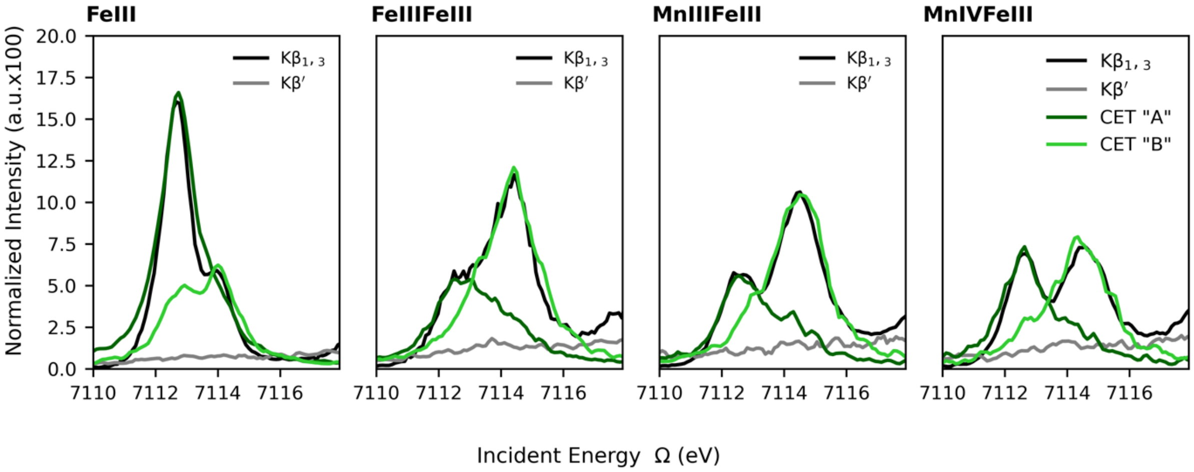

Figure S3. Overlays of Fe CEE vs CET within the pre-edge region along the incident energy axis.

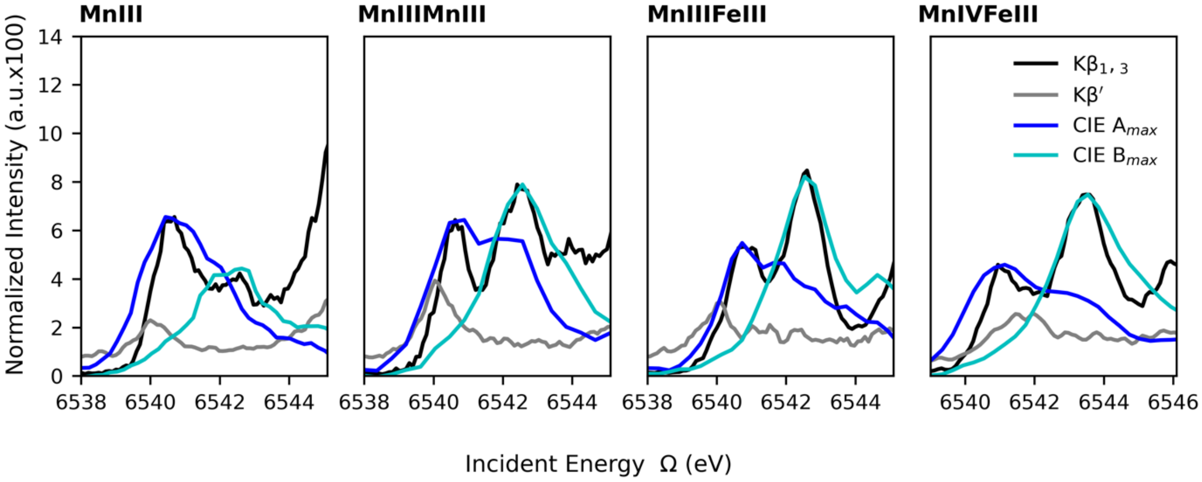

Figure S4. Overlays of Mn CIE vs CEE within the pre-edge region along the incident energy axis. CIE data was shifted so CIE A matches the chosen constant incident energy.

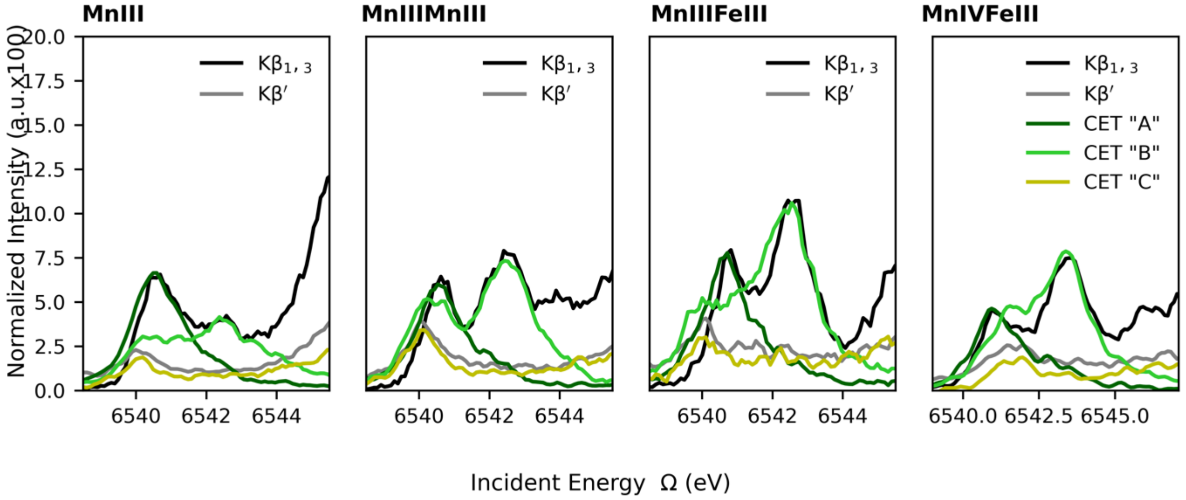

Figure S5. Overlays of Mn CEE vs CET within the pre-edge region in the incident energy axis.

Table S4. 1s3p RIXS instrumental and lifetime broadenings for Fe and Mn 1s3p RIXS

	Fe	Mn
Experimental broadening *	1.1 eV	1.2 eV
Incident monochromator broadening	0.2 eV	0.2 eV
Emission broadening**	1.08 eV	1.18 eV
Γ_{1s} (CET broadening)***	1.15 eV	1.11 eV
Γ_{3p} (CIE broadening)***	1.4 eV	1.2 eV
Γ_{APP} [2, 3]	0.88 eV	0.81 eV
fv (HERFD broadening)[3]	0.93 eV	0.86 eV
CIE total	1.64 eV	1.71 eV
CET total	1.16 eV	1.12 eV

** Average FWHMs of elastic peaks in each crystal*

***Emission broadening computed from the deconvolution of experimental broadening and incident broadening.*

****Values taken from Hephaestus software.[4]*

Pre-edge XAS Simulations

In order to assess which intermediate states contribute to the RIXS planes, the pre-edge XAS spectrum has been simulated for Mn(III) and Mn(IV). Simulations utilized a reduction of the Slater integrals to 65% of their atomic values and an octahedral ligand field with 10Dq equal to 2.0 eV. The change in orbital population upon core excitation is also given to confirm the assignment of transitions. The pre-edge intensities are computed as quadrupole strengths.

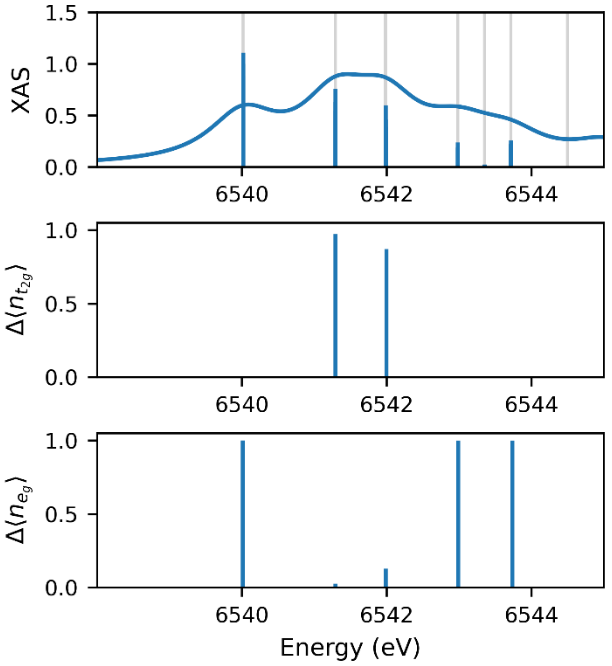

Figure S6. Pre-edge XAS simulation of Mn(III). (top) Simulation of pre-edge showing transitions that yield spectral intensity (blue sticks) together with all quintet states (gray lines) arising within the shown energy range. Orbital character of transitions is given by the difference in the expectation values of the number operator between ground and core-excited states. Values are summed over the t_{2g} (middle) and e_g (bottom) orbital sets.

Table S5. $3d^5$ states arising from the $1s^13d^5$ core-excited configuration of Mn(III)

3d Term	Energy (eV)	Quadrupole Intensity
^6A	6540	Yes
^4T	6541.3	Yes
^4T	6542.0	Yes
^4T	6543	Yes
^4T	6543.3	No
^4E	6543.7	Yes
^4T	6544.5	No
^4A	6545	Yes
^4T	6545.7	No
^4T	6546.4	No

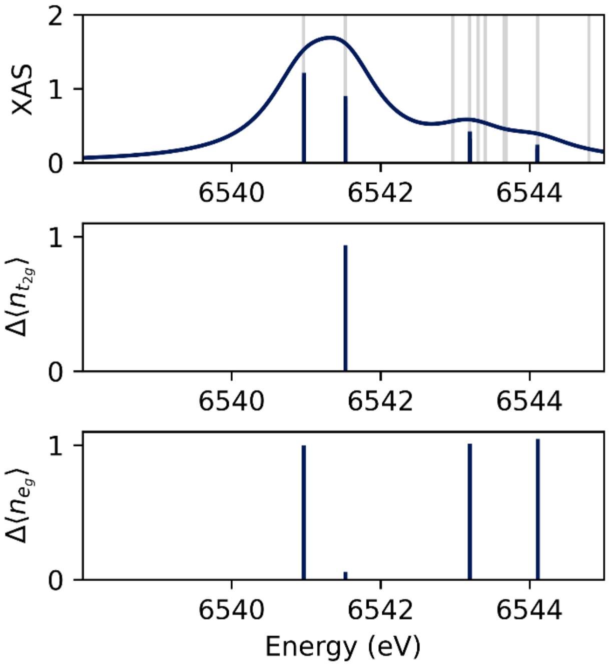

Figure S7. Pre-edge XAS simulation of Mn(IV). (top) Simulation of pre-edge showing transitions that yield spectral intensity (blue sticks) together with all quintet states (gray lines) arising within the shown energy range. Orbital character of transitions is given by the difference in the expectation values of the number operator between ground and core-excited states. Values are summed over the t_{2g} (middle) and e_g (bottom) orbital sets.

Table S6. $3d^4$ states arising from the $1s^13d^4$ core-excited configuration of Mn(IV)

3d Term	Energy (eV)	Quadrupole Intensity
^5E	6541	Yes
^3T	6541.5	Yes
^5T	6543.0	No
^3E	6543.2	Yes
^3T	6543.3	No
^3T	6543.4	No
^3E	6543.7	No
^3E	6544.1	Yes
^3T	6544.8	No

MnIII CET and Jahn-Teller distortion

For Mn(III) cases, out of all possible excited states, the ligand field dependency can be monitored by the $^5T(^4T)$ intermediate state, which can be isolated experimentally and computationally, and compared with the corresponding Tanabe Sugano diagram for d^5 in the 1s3p RIXS picture (with d^{n+1} excited states). However due to the inclusion of Jahn Teller effects a more accurate correlation is done when including such distortion.

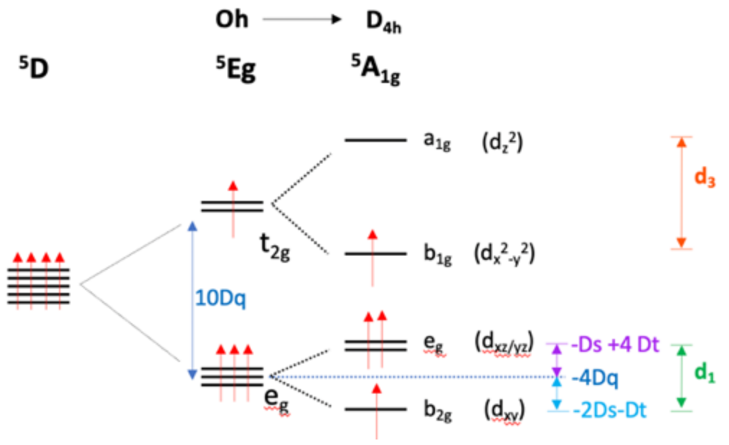

Figure S8. Oh and Jahn Teller 3d energy levels .

Figure S9 shows the result of including various Jahn-Teller distortions in the 1s3p RIXS spectrum. All Mn(III)-containing models have a compressed octahedron as shown in the diagram above. Parameters used were obtained from reported data from Brunold *et al.* [Ref 46 in manuscript] on from DFT calculations in MnIIIMnIII model, and from current DFT calculations runed during this experiment for TD-DFT calculations of the pre-edges:

JT: Reported[1] tetragonal compressed: d1 = dxy- dxz/dyz = ~0.2 eV; d3 = dx2-y2 – dz2 = 0.4 eV
JT1: DFT tetragonal compressed: d1 = dxy- dxz/dyz = ~0.4 eV; d3 = dx2-y2 – dz2 = 1.52 eV

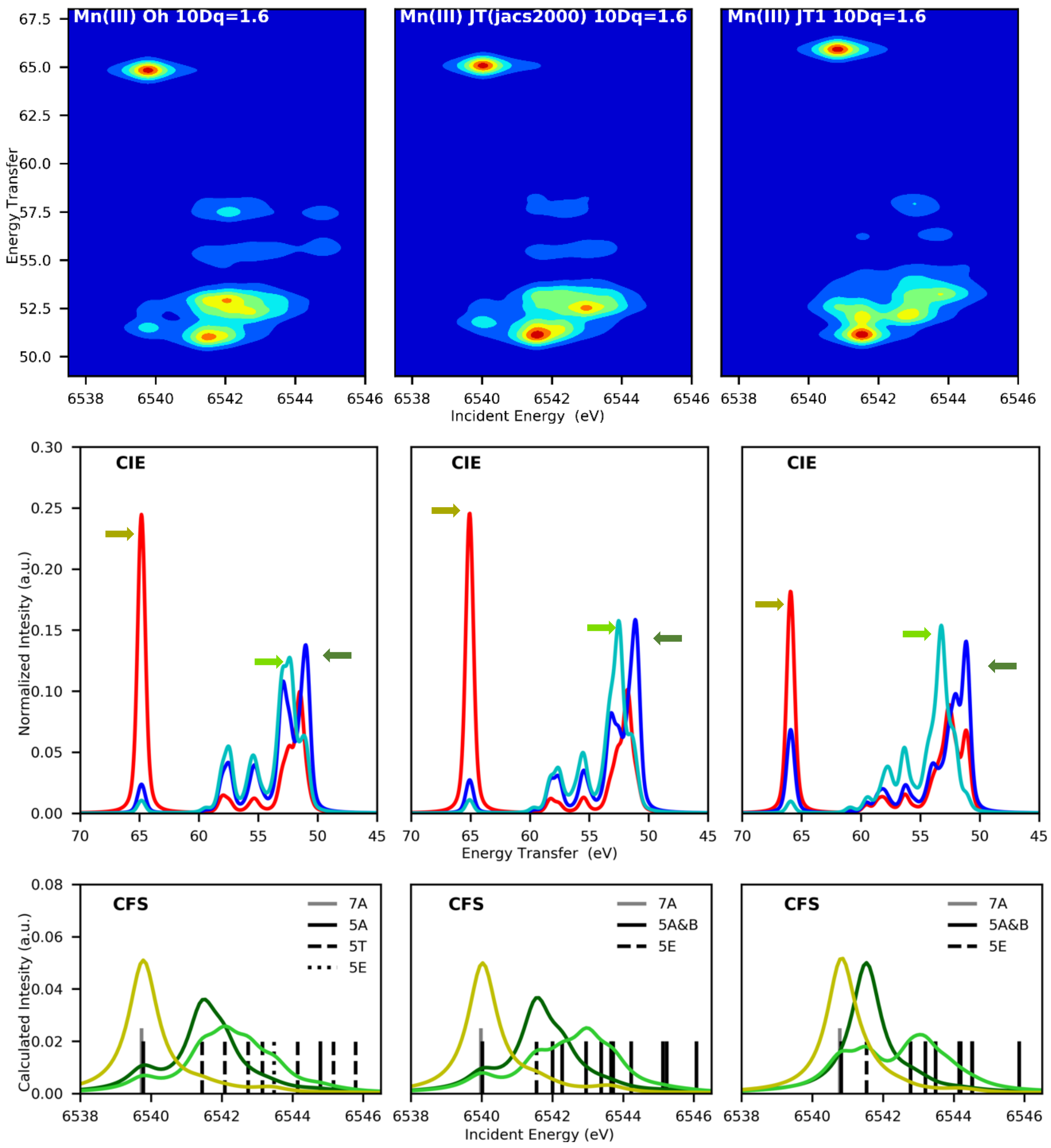

Figure S9. Mn(III) simulated RIXS map for O_h and D_{4h}.

MnIV CET and Ligand Field effects

Although we only present experimental data on one Mn(IV) compound (MnIVFeIII), the ligand field dependency is also expected to have a direct correlation with the CET cuts. For the Mn(IV) d^{n+1} excited states, the d^4 Tanabe Sugano diagram show that from all the possible 1s3p excited states, the ^4T state with the local term ^3T in the $3d^{n+1}$-manifold has the largest ligand field dependency. This intermediate state can be isolated experimentally and computationally.

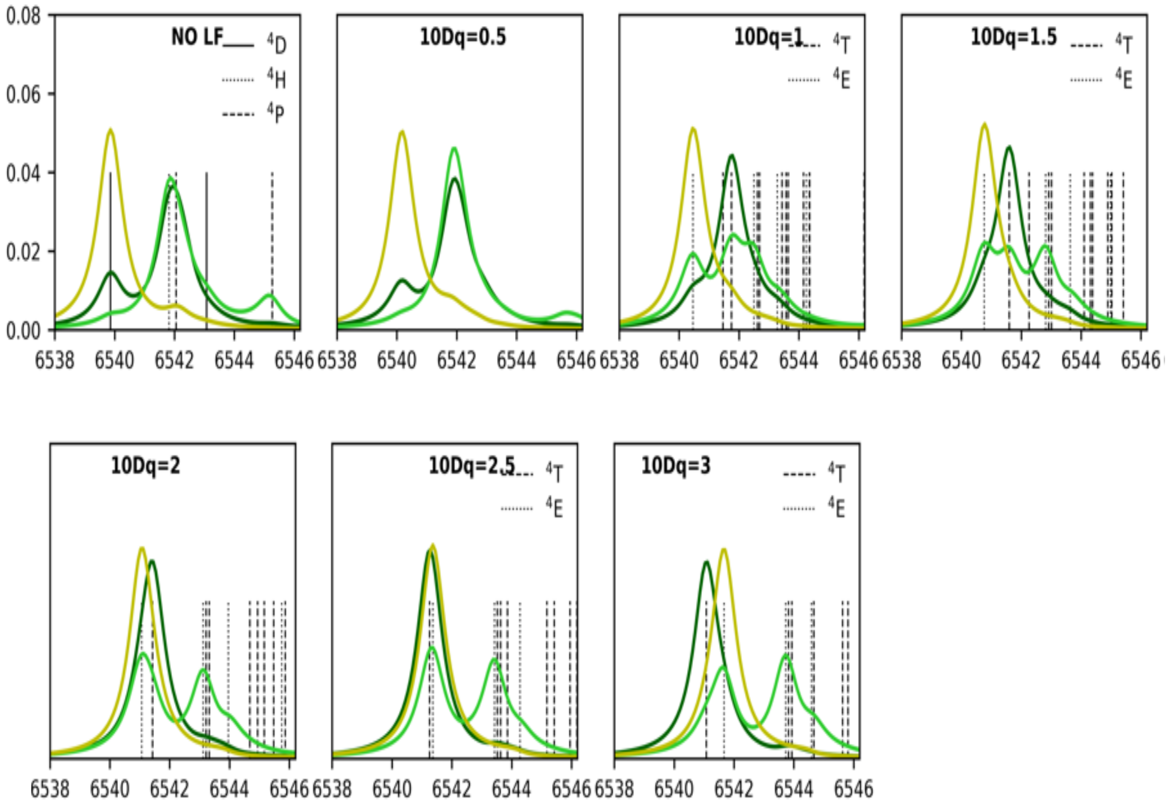

Figure S10. Calculated CET for MnIV varying LF 10Dq between 0 and 3.

Reduction of the Slater Condon Parameters Scaling factor for Mn(IV).

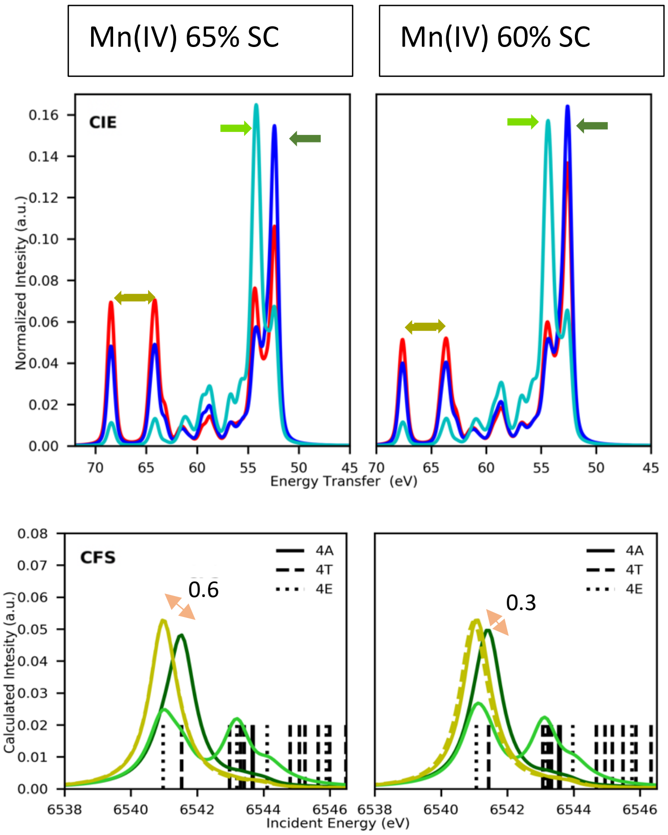

Figure S11. Influence of covalency in the energy of all final states in CFS by using 60 and 65 % scaling factor of the Slater Condon Integrals (SC).

REFERENCES

(1) Brunold, T. C.; Gamelin, D. R.; Solomon, E. I. Excited-state exchange coupling in bent Mn (III)– O– Mn (III) complexes: Dominance of the π/σ superexchange pathway and its possible contributions to the reactivities of binuclear metalloproteins. *Journal of the American Chemical Society* **2000**, *122* (35), 8511-8523.

(2) Glatzel, P.; Bergmann, U. High resolution 1s core hole X-ray spectroscopy in 3d transition metal complexes—electronic and structural information. *Coordination chemistry reviews* **2005**, *249* (1-2), 65-95.

(3) Glatzel, P.; Weng, T.-C.; Kvashnina, K.; Swarbrick, J.; Sikora, M.; Gallo, E.; Smolentsev, N.; Mori, R. A. Reflections on hard X-ray photon-in/photon-out spectroscopy for electronic structure studies. *Journal of Electron Spectroscopy and Related Phenomena* **2013**, *188*, 17-25. DOI: https://doi.org/10.1016/j.elspec.2012.09.004

(4) Ravel, B.; Newville, M. ATHENA, ARTEMIS, HEPHAESTUS: data analysis for X-ray absorption spectroscopy using IFEFFIT. *Journal of synchrotron radiation* **2005**, *12* (4), 537-541.
